# Controlled Bioactive Delivery Using Degradable Electroactive Polymers

**DOI:** 10.1021/acs.biomac.2c00516

**Published:** 2022-06-24

**Authors:** Mark D. Ashton, Patricia A. Cooper, Sofia Municoy, Martin F. Desimone, David Cheneler, Steven D. Shnyder, John G. Hardy

**Affiliations:** † Department of Chemistry, Faculty of Science and Technology, Lancaster University, Bailrigg, Lancaster LA1 4YB, U.K.; ‡ Institute of Cancer Therapeutics, School of Pharmacy and Medical Sciences, Faculty of Life Sciences, University of Bradford, Bradford BD7 1DP, U.K.; § Instituto de Química y Metabolismo del Fármaco (IQUIMEFA), Facultad de Farmacia y Bioquímica, Consejo Nacional de Investigaciones, Científicas y Técnicas (CONICET), Universidad de Buenos Aires, Junín 956, Piso 3° (1113), Buenos Aires 1113, Argentina; ∥ Department of Engineering, Faculty of Science and Technology, Lancaster University, Bailrigg, Lancaster LA1 4YW, U.K.; ⊥ Materials Science Institute, Lancaster University, Bailrigg, Lancaster LA1 4YB, U.K.

## Abstract

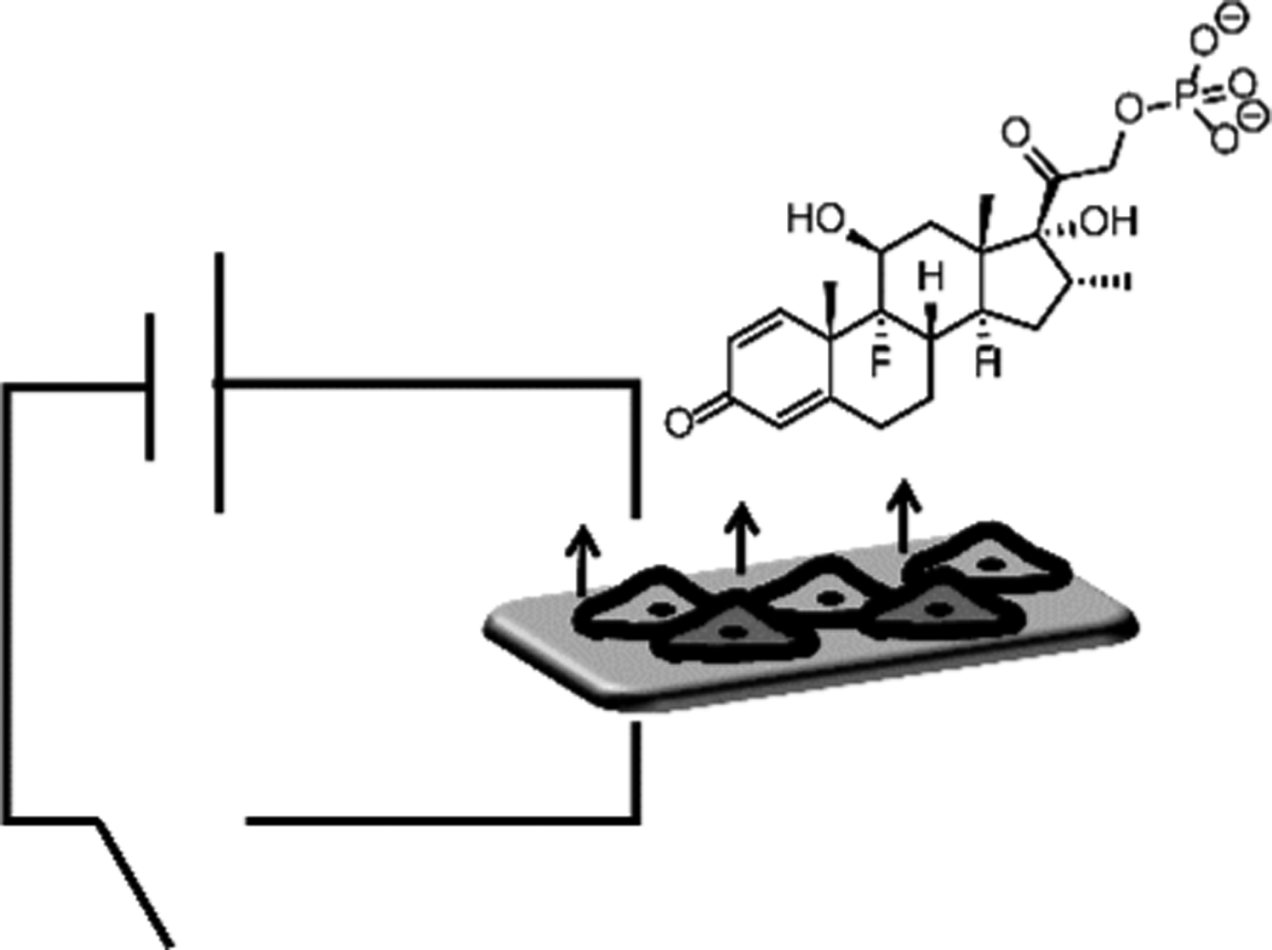

Biomaterials capable of precisely controlling the delivery of agrochemicals/biologics/drugs/fragrances have significant markets in the agriscience/healthcare industries. Here, we report the development of degradable electroactive polymers and their application for the controlled delivery of a clinically relevant drug (the anti-inflammatory dexamethasone phosphate, DMP). Electroactive copolymers composed of blocks of polycaprolactone (PCL) and naturally occurring electroactive pyrrole oligomers (e.g., bilirubin, biliverdin, and hemin) were prepared and solution-processed to produce films (optionally doped with DMP). A combination of in silico/in vitro/in vivo studies demonstrated the cytocompatibility of the polymers. The release of DMP in response to the application of an electrical stimulus was observed to be enhanced by ca. 10–30% relative to the passive release from nonstimulated samples in vitro. Such stimuli-responsive biomaterials have the potential for integration devices capable of delivering a variety of molecules for technical/medical applications.

## Introduction

The development/application of novel drug delivery systems capable of precisely controlling the delivery of their payloads is an area of intense current research interest as the importance of personalized medicine has been understood.^1−4^ Such systems potentially enable spatiotemporally controlled delivery, for example, maintaining a therapeutically effective level of a drug, minimizing unwanted side effects, and thereby enhancing treatment efficiency.^5,6^

Stimuli-responsive materials have been used in the development of drug delivery systems (DDSs) that can control drug release using endogenous stimuli (e.g., enzymes, pH, etc.)^7−9^ and exogenous stimuli (e.g., electric fields,^10,11^ infrared (IR),^12,13^ light,^14,15^magnetism,^16,17^ radiation,^18^ temperature,^19^ and ultrasound,^16^ to name a few),^20^ with a model DDS allowing control of the location and dosage of the drug,^8,21^

Electroactive polymers (EAPs) are a class of stimuli-responsive polymers with a variety of technical and medical applications, with popular nondegradable examples including, but not limited to, polyaniline, polypyrrole, and poly(3,4-ethylenedioxythiophene) (PEDOT).^11,22−24^ The integration of nondegradable EAPs in medical devices, such as electrodes for sensing/stimulation, offers opportunities to optimize tissue–electrode interactions, such as their mechanical properties (minimizing mechanical mismatch) or the impedance of the electrodes (enhancing the longevity of function).^25−27^

The development of degradable/transient EAPs^28−33^ offers opportunities for the production of materials for short- to medium-term applications, including (but not limited to): tissue scaffolds for tissue engineering and regenerative medicine wherein the scaffold would eventually degrade and ideally be replaced by healthy functional tissue,^10,34^ and system capable of controlled release of agrochemicals/biologics/drugs/fragrances followed by their degradation.^28,35^

The electroactive nature of EAPs facilitates their application as DDSs that respond to electrical stimuli (which function by various mechanisms including actuation, charge passage, redox switching),^25^ potentially enabling precise spatiotemporal control of the amount of drug at any time. Nondegradable EAP-based DDSs can be useful electrode coatings (e.g., for delivery of anti-inflammatories to minimize inflammation in the proximity of implantation of the electrodes) either in response to an electrical signal delivered by wire^25,36,37^ or indeed remotely/wirelessly.^38−40^

The first examples of degradable EAP-based DDSs were based on electroactive oligomers of aniline combined with nonelectroactive blocks,^29,41^ and since that time, other examples have been reported in the literature incorporating electroactive oligomers (typically oligoanilines).^42,43^ The literature precedent on the toxicity of oligoanilines^28,38,44^ motivates the investigation of biomaterials incorporating other electroactive moieties, for example, oligopyrroles/porphyrins, which are being developed for a multitude of technical and medical applications.^45−50^

Polycaprolactones (PCLs) are a family of U.S. Food and Drug Administration (FDA)-approved nontoxic polymers that are biodegradable over extended periods of time.^51^ PCLs are commonly used in biomaterials as a result of being biodegradable, biocompatible, and easily processed via melt/solution processing into various material morphologies (e.g., films, fibers, foams, particles, etc.).^52−55^ Here, we report the development of degradable EAPs employing a combination of derivatives of PCL and naturally occurring electroactive pyrrole oligomers (e.g., bilirubin, biliverdin, or hemin)^56−58^ that can be prepared at gram scale in simple and scalable syntheses with simple purifications. The polyesters were solution-processed to produce films (optionally doped with different molecules) that were characterized by a variety of techniques. The polymers have been designed to eventually degrade to low-molecular-weight species readily clearable by the renal system.^28,59^ A combination of in silico, in vitro, and in vivo studies was used to assess the cytocompatibility of the polymers. The release of the anti-inflammatory drug (DMP, often dosed multiple times in the clinic)^60^ from DMP-doped polymer films in the absence/presence of electrical stimuli was assessed via UV spectroscopy.

## Experimental Section

### Materials

All compounds were synthesized using reagent-grade starting materials purchased from Sigma-Aldrich, Fisher Scientific, and Alfa Aesar, and used as received. The purity of all products was verified via thin-layer chromatography (TLC) using Fluka analytical TLC plates (stationary phase, 60 Å pore size silica; thickness, 0.2 mm) before undergoing any further analysis.

### Synthesis of Polymer 1 (Copolymer of PCL Diol 530 + Hemin)

Hemin (0.65 g, 1 mmol), PCL diol 530 (0.53 g, 1 mmol), DIC (5 mL), and DMAP (10 mg, 0.67 mmol) were dissolved in NMP (7 mL). The solution was stirred for 72 h under nitrogen at room temperature affording a black solution. The reaction mixture was taken up in DCM (10 mL); washed with a 1 M aqueous solution of HCl (2 × 25 mL), a 1 M aqueous solution of NaOH (2 × 25 mL), and brine (25 mL); dried over MgSO_4_; and evaporated under reduced pressure. The resulting solution was added dropwise to a stirring solution of cold (0 °C) diethyl ether (500 mL), affording a black solid, which was isolated by vacuum filtration and dried in a vacuum oven for 72 h, in a yield of 64%, 756 mg. UV–vis (DCM): λ_max_ (ε): 396 nm, IR (−OH) 3371 cm^–1^, (−C=O) 1731 cm^–1^, (−C–O) 1111 cm^–1^, GPC (THF)—4.164 × 10^5^ g·mol^–1^

### Synthesis of Polymer 2 (Copolymer of PCL Diol 2000 + Hemin)

Hemin (0.65 g, 1 mmol), PCL diol 2000 (2.03 g, 1 mmol), DIC (5 mL), and DMAP (10 mg, 0.67 mmol) were dissolved in NMP (7 mL) under nitrogen. The solution was stirred for 72 h under nitrogen at room temperature affording a black solution. The resulting solution was added dropwise to a stirring solution of cold (0 °C) diethyl ether (500 mL) affording a black solid, which was isolated by vacuum filtration and dried in a vacuum oven for 72 h, in a yield of 67%, 1.52 g. UV–vis (DCM): λ_max_ (ε): 387 nm, IR (−OH) 3362 cm^–1^, (−C=O) 1736 cm^–1^, (−C–O) 1129 cm^–1^, GPC (THF)—1.449 × 10^6^ g·mol^–1^

### Synthesis of Polymer 3 (Copolymer of PCL Triol 900 + Hemin)

Hemin (0.975 g, 1.5 mmol), PCL triol 900 (0.90 g, 1 mmol), DIC (5 mL), and DMAP (10 mg, 0.67 mmol) were dissolved in NMP (7 mL). The solution was stirred for 72 h under nitrogen at room temperature affording a black solution. The reaction mixture was taken up in DCM (10 mL); washed with a 1 M aqueous solution of HCl (2 × 25 mL), a 1 M aqueous solution of NaOH (2 × 25 mL), and brine (25 mL); dried over MgSO_4_; and evaporated under reduced pressure. The resulting solution was added dropwise to a stirring solution of cold (0 °C) diethyl ether (500 mL) affording a black solid, which was isolated by vacuum filtration and dried in a vacuum oven for 72 h, in a yield of 35%, 630 mg. UV–vis (DCM): λ_max_ (ε): 395 nm, IR (−OH) 3381 cm^–1^, (−C=O) 1737cm^–1^, (−C–O) 1126 cm^–1^, GPC (THF)—2.259 × 10^4^ g·mol^–1^

### Synthesis of Polymer 4 (Copolymer of PCL Diol 2000 + Bilirubin)

Bilirubin (0.585 g, 1 mmol), PCL diol 2000 (2.04 g, 1 mmol), DIC (5 mL), and DMAP (10 mg, 0.67 mmol) were dissolved in NMP (7 mL) under nitrogen. The solution was stirred for 72 h under nitrogen at room temperature affording a black solution. The resulting solution was added dropwise to a stirring solution of cold (0 °C) diethyl ether (500 mL) affording a yellow solid, which was isolated by vacuum filtration and dried in a vacuum oven for 72 h, in a yield of 64%, 1.649 g. UV–vis (DCM): λ_max_ (ε): 413 nm, IR (−OH) 3376 cm^–1^, (−C=O) 1740 cm^–1^, (−C–O) 1143 cm^–1^, GPC (THF)—1.222 × 10^6^ g·mol^–1^

### Synthesis of Polymer 5 (Copolymer of PCL Diol 2000 + Biliverdin)

Biliverdin hydrochloride (0.03 g, 1 mmol), PCL diol 2000 (0.106 g, 1 mmol), DIC (5 mL), and DMAP (10 mg, 0.67 mmol) were dissolved in NMP (7 mL) under nitrogen. The solution was stirred for 72 h under nitrogen at room temperature affording a black solution. The resulting solution was added dropwise to a stirring solution of cold (0 °C) diethyl ether (500 mL) affording a blue solid, which was isolated by vacuum filtration and dried in a vacuum oven for 72 h, in a yield of 70%, 105 mg. UV–vis (DCM): λ_max_ (ε): 377 nm, IR (−OH) 3384 cm^–1^, (−C=O) 1753 cm^–1^, (−C–O) 1135 cm^–1^, GPC (THF)—1.449 × 10^6^ g·mol^–1^

### Fourier Transform Infrared (FTIR) Spectroscopy

All spectra were recorded using an Agilent Technologies Cary 630 FTIR instrument (Agilent Technologies Ltd., Cheadle, U.K.) at a resolution of 1 cm^–1^, and an average of 16 scans were taken.

### Nuclear Magnetic Resonance (NMR) Spectroscopy

^1^H NMR (400 MHz) was attempted on all synthesized compounds using a Bruker AVANCE III 400 NMR spectrometer with a tetramethylsilane (TMS) internal standard in deuterated solvents. Chemical shift (δ) values were recorded in parts per million (ppm), and the peaks were labeled as singlet (s), doublet (d), triplet (t), quartet (q), or multiplet (m) where possible.

### Gel Permeation Chromatography (GPC)

GPC data were obtained using a Shimadzu GPC/SEC spectrometer equipped with a Phenomenex Phenogel column. GPC grade THF was selected as the mobile phase and using a flow rate of 1.0 mL/min at 40 °C. A polystyrene standard of Mw 30,000 Da and internal standards ranging from Mw 580 to 325,600 were used to normalize the data and construct a calibration curve. All standards and samples were fully dissolved in GPC-grade THF at a concentration of 1 mg/mL and left overnight before being filtered using PTFE filters prior to analysis.

### Film Preparation

Polymer (20 mg) was dissolved in 1 mL of THF and drop-cast onto a PTFE tile until the liquid was evenly distributed across the tile. Excess THF was allowed to evaporate, leaving a film on the surface of the tile, which was dried in a vacuum oven for 24 h at room temperature. The films were cut using a metal ruler affording a uniform film, which could be readily removed from the PTFE tile (Fisher Scientific Loughborough, U.K.).

### Profilometry

Profilometry was carried out using a Mitutoyo Surftest sj-400 contact profilometer. A step change was utilized to show the change in thickness between the glass slide and the film on the slide. Data analysis was carried out with the software provided by the manufacturer, which allowed the determination of the thickness and roughness of the films (errors are expressed as standard deviation). Each film was analyzed in three places to give an average roughness reading, measuring at a speed of 0.05 mm/s over a sample length of 0.8 mm. The surface roughness parameters were analyzed and reported in accordance with the ISO 25178 series. The average roughness (*R*_a_) is the arithmetic average of the deviation from the mean line and is the most used international parameter of roughness. The average height difference between the five highest peaks and the five lowest valleys (R_Z_DIN) was determined in accordance with DIN 4768/1 as specified by the Deutsches Institut für Normung.

### Water Contact Angle Measurements

Measurements were carried out with a high-speed contact angle measurement device (Ossila Contact Angle Goniometer, Sheffield, U.K.) and analyzed via ImageJ with the plugin drop analysis. Images of a drop of deionized water (2 μL) laid on the surface of the samples were recorded at a frame rate of 360 frames per second, and the contact angles for the droplets were recorded after 3 s of contact with the film. The reported values are the average of at least three measurements at different positions on a film.

### Thermogravimetric Analysis (TGA)

TGA was carried out on all samples using a Netzsch TG 209 FT Tarsus thermogravimetric analyzer on samples of mass 5–10 mg with a ramp rate of 10 °C min^–1^ under a continuous flow of nitrogen. Below 200 °C, the weight loss observed in the polymers was attributed to the evaporation of residual solvents.

### Mechanical Studies

Measurements of film thicknesses were recorded prior to mechanical testing via profilometry (described above), and these values were used in the calculation of elastic modulus, tensile strength, and the strain at failure. The mechanical properties of polymer films were ascertained using an Instron 10 N static load cell, 2.5 mm Clevis pin with 6 mm adapter (Type OOf & Of), 2530-10N, with bespoke clamps on an Instron 3345 single column mechanical tester (5 kN (1125 lbf) capacity, 1123 mm (44.2 in) vertical test space, 1383 mm (54.4 in) vertical test space (Extra-height model)). Reading parameters: force measurement accuracy (±0.5% of reading down to 1/200 of the load cell capacity), displacement measurement accuracy (0.15% of displacement), and data acquisition rate (0.5 kHz simultaneous on force, displacement).

### Ultraviolet–visible (UV–Vis) Spectroscopy

UV–vis studies on films on quartz slides were carried out using an Agilent Technologies Cary 60 UV–vis spectrometer.

### Voltammetry

Voltammetry experiments were carried out using an EmStat 3+ potentiostat with PSTrace 4.7 software (PalmSens Houten, Netherlands) at ambient temperature. The cell comprised a three-electrode system with a Ag/AgCl reference electrode, a gold counter electrode, and a glassy carbon working electrode (films of polymers coating the glassy carbon electrodes were prepared by drop-casting the polymers dissolved in THF followed by drying for 24 h in a vacuum oven at room temperature). Phosphate-buffered saline (PBS, 0.01 M, pH 7.4, 4 mL) was used as the electrolyte, with a scan rate of 0.05 V/s between −1 and 1 V.

### Conductivity Determination

The conductance of films was measured in accordance with protocol IPC-TM-650, number 2.5.17.2 described by the Institute for Interconnecting and Packaging Electronic Circuits. Films supported on glass slides were examined by chronoamperometry using a Keithley 2612B source meter (Tektronix, Beaverton, US). Chronoamperometric measurements were made with a two-point probe system (copper alligator clips), by connecting counter and reference electrodes together. Briefly, two thin strips of adhesive-backed copper tape (Ted Pella, Inc., Redding, CA) were attached to the films, parallel to one another, separated by a distance of 0.5 cm. The working and counter electrodes were clipped on the strips of copper tape, and the current was measured for 30 s during a potential step experiment at 10 V. The electrodes were moved to different positions after each measurement, and the current passed was recorded at three different positions. The resistance (*R*, Ω) of the films was determined in accordance with eq 1
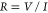
1The resistivity (Ω cm^–1^) of the films was determined in accordance with eq 2
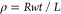
2where *w* corresponds to the width of the film in cm (2.5 cm); *t* corresponds to the thickness of the film in cm (as determined *via* profilometry); and *L* corresponds to the length of the film in cm (0.5 cm). The conductivity (S cm^–1^) of the films was determined in accordance with eq 3
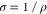
3

### In Silico Studies

In silico toxicity screening was carried out using Derek/Sarah Nexus, and forced degradation predictions were carried out using Zeneth (Derek Nexus: v. 6.0.1, Nexus: 2.2.2; Sarah Nexus: v. 3.0.0, Sarah Model: 2.0; Zeneth: v. 8.1.1) supplied by Lhasa Limited, Leeds, U.K.

### In Vitro Cell Culture Studies

Polymer films were sterilized prior to cell culture experiments, submerged for 24 h in a solution of ethanol:water (7:3), and dried in a desiccator before use. NIH3T3 fibroblast cell line was cultured using Dulbecco’s modified Eagle’s medium (DMEM) with 10% fetal calf serum, 1% penicillin–streptomycin, and 0.25 μg/mL Fungizone, under an atmosphere of 5% CO_2_ at 37 °C. For experiments, cells were harvested using a trypsin–EDTA solution and the number of viable cells was counted with a Neubauer camera after staining with trypan blue. Fibroblast cells (1 × 10^4^ cells per well) were added on top of each film or to the wells of a 24-well plate (in the case of control cells), with 0.5 mL of cell culture medium and kept in a humidified 5% CO_2_ atmosphere at 37 °C. The cell metabolic activity was measured by the colorimetric 3-(4,5-dimethyl-thiazol-2-yl)-2,5-diphenyltetrazolium bromide (MTT) assay after 1–7 days. Briefly, the cell culture medium was removed and a solution of MTT in PBS 1× (5 mg/mL) was added to the cells and incubated at 37 °C for 4 h. Then, the MTT solution was discarded, films were washed with PBS 1X and absolute ethanol was added. Finally, the absorbance of the purple solution was measured at 570 nm and results were expressed as mean ± SD from triplicate experiments.^61−63^

### In Vivo Implantation Studies

All animal work was carried out in accordance with the UK Animal Scientific Procedures Act and approved by the University of Bradford Animal Welfare and Ethical Review Body under project license PBA7ACB920 issued by the UK Home Office.

Material for transplantation was prepared using a standard operating procedure in a class II biological safety cabinet to maintain sterility once prepared. Samples were cut into 10 mm × 4 mm strips using a double-edged razor blade, then placed in a separate well of a six-well cell culture plate, and immersed in 70% ethanol for 60 s to sterilize them. They were allowed to air-dry and then placed in a labeled well of a fresh six-well plate and stored in the dark at room temperature until ready to use.

Female BALB/c mice (Envigo, U.K.) aged 8 weeks were used in the study. The animals were maintained in the animal facilities of the University of Bradford. The mice were housed in cages not exceeding the numbers according to UK Home Office regulations and were provided with bedding, nesting material, and perspex housing. They were provided with food (Teklad 2018 diet, Envigo, U.K.) and water *ad libitum*. A 12-h light-on light-off cycle was observed, and the animal holding room temperature was maintained at 21 °C with 50% humidity.

The mice were anesthetized and maintained under isoflurane inhalation anesthesia during the surgical procedure, with the mice placed on a heating pad maintained at 37 °C during recovery from anesthesia. A 2.5 cm × 1 cm strip on each dorsal flank (above the hips) was shaved with electric clippers and a horizontal incision of 5–6 mm was made at the lower end of the shaved area and a pocket between the skin and the abdominal wall was made using tweezers. Using tweezers, the samples were inserted into the pocket, and the incision was sealed with Histoacryl tissue glue (Miller Medical Supplies, U.K.). For sham surgical controls, no material was inserted in the pocket, but the incision was again sealed with tissue glue.

The animals were monitored three times a week for any deleterious effects including signs of inflammation at the implantation site, and their bodyweight was measured. On days 7, 28, and 70, a digital image of each sample (sham and nonsham) in situ was taken, and 10 μL of blood was collected from the tail vein to produce blood films as detailed below. On each sample, day 9 mice were sacrificed by cervical dislocation, giving three implants per material or sham for each time point. With a 3–4 mm margin around the implant, the samples sandwiched between the skin and abdominal wall were excised and fixed in neutral buffered formalin before routine processing for paraffin embedding for subsequent histological examination.

Sections (5 μm thick) were taken for each paraffin block and stained with Harris’s hematoxylin and eosin. The sections were examined at ×400 magnification under bright-field illumination on a Leica DMLB microscope, and the thickness in mm of the granulation tissue surrounding the implant was measured in 10 high-powered fields for each sample and the mean thickness was determined.

For blood films, a 5 μL drop of blood was placed at one end of each of two labeled microscope slides per sample, and then a smear was made across the slide using the edge of another slide. This slide was allowed to air-dry and then stained with Giemsa to highlight the different white blood cell types. Films were then examined at ×400, and 200 white blood cells were counted across the film, with the numbers of lymphocytes, neutrophils, and other white blood cells (monocytes, eosinophils, and basophils) recorded.

### Drug Delivery Studies In Vitro

Chronoamperometric studies were completed using a PalmSens EmStat 3+ potentiostat using PSTrace 4.7 software (supplied by Alvatek, Tetbury, U.K.). The cell comprised a three-electrode system with an Ag/AgCl reference electrode, a gold counter electrode, and a glassy carbon working electrode in PBS (4 mL). Each polymer (3 mg) and DMP (1 mg) were dissolved in 1 mL of THF and drop-cast on the glassy carbon electrode, before being dried for 24 h in a vacuum oven at room temperature and added to the cell. Before each experiment, the initial potential of the cell was held at 0 V for 10 s before alternating between the high potential of around 0.7 V and the low potential at approximately −0.5 V. The current was measured at 1 mV intervals using a 50 mV·s^–1^ scan rate with each stimulation lasting 62 s. After stimulation of the film, the cell was allowed to rest for 14 min to allow the released drug to equilibrate in the PBS solution. After allowing the drug to equilibrate in solution post-stimulation, a 10 μL aliquot was taken from the electrolyte solution and diluted with 100 μL of PBS before being frozen prior to analysis. Passive release controls were run in parallel with electrically stimulated samples. Samples were prepared in low UV Corning 96 well, clear-bottom, flat-base, polystyrene microplates. Absorbance measurements were taken in triplicate using a Flexstation 3 plate reader (Molecular Devices, San Jose) at 242 nm (DMP).

## Results and Discussion

**Figure 1:**
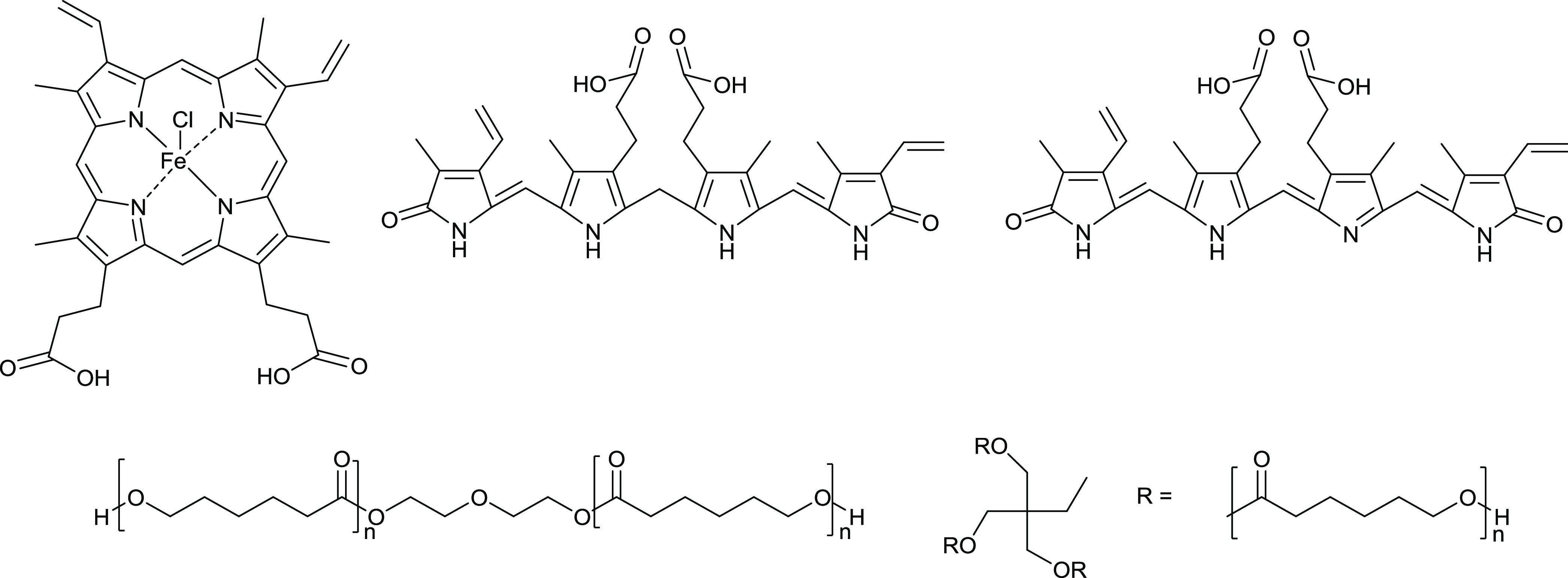
Chemical structures of EAP building blocks, Hemin (top left), Bilirubin (top middle), Biliverdin (top right), PCL diol (middle left), and PCL triol (middle right).

The EAPs reported herein are block copolymers prepared by esterification of PCL derivatives terminated with alcohols and naturally occurring electroactive pyrrole oligomers displaying carboxylic acids (bilirubin, biliverdin, or hemin), as depicted in Figure 1. Simple, scalable Steglich esterifications (Scheme S1) were employed to produce polyesters in yields between 56 and 70% in a single step from commercially available starting materials without column chromatography. The color of the polymers was determined by the nature of the pyrrole oligomer incorporated; hemin-containing polymers **1–3** were black, whereas bilirubin-containing polymer **4** was orange, and biliverdin-containing polymer **5** was blue.

The success of the condensation polymerization was confirmed by FTIR spectroscopy (Figures S1–S11), with peaks at 716 cm^–1^ (characteristic of the aromatics present in the pyrrole oligomers), 1111 cm^–1^ (characteristic of the ether bonds present in the PCL derivatives), 1731 cm^–1^ (characteristic of ester bonds), 2858 and 2927 cm^–1^ (characteristic of C–H bonds), and 3300–3400 cm^–1^ (characteristic of O–H from traces of water). Iron (III) protoporphyrin derivatives (e.g., the hemin-containing polymers) are not possible to be characterized by NMR due to the paramagnetic iron atom coordinating to various surrounding molecules (e.g., solvents);^64^ the presence of solvent molecules results in an intermediate exchange of hemin chloride between high-spin d^5^ and d^6^ coordinate, and this change in spin state causes broadening of the NMR peaks, whereas the NMR spectra of the bilirubin-/biliverdin-containing polymers (Figures S12 and S13) showed peaks characteristic of both the PCL (1–4 ppm) and oligopyrrole units (aromatic region) of the NMR spectra, integration of which confirmed that the diol and diacid blocks were present in a 1:1 ratio, whereas for **3** incorporating the triol, the ratio was 2:3, triol:diacid (adjusted during synthesis to account for the extra hydroxyl group on the triol). Gel permeation chromatography (GPC) data also confirmed the successful polymerization with polydispersities between 1.0 and 1.25 (Table 1, Figures S14–S18). Polymers incorporating PCL diol 2000 (polymers **2**, **4**, and **5**) had Mw values of approximately 10^6^ Da, whereas those incorporating PCL diol 530 or PCL triol 900 (polymers **1** and **3**, respectively) had lower Mw and higher polydispersities, likely due to the effect of steric hindrance during synthesis.^65^

**Table 1:** Properties of Polymers **1**–**5** and Films Composed Thereof, and Granulation Tissue Depth after Implantation in Mice^a^

polymer	1 (PCL diol 530 + hemin)	2 (PCL diol 2000 + hemin)	3 (PCL triol 900 + hemin)	4 (PCL diol 2000 + bilirubin)	5 (PCL diol 2000 + biliverdin)
Mw (g/mol)	4.16 × 10^5^	1.45 × 10^6^	2.26 × 10^4^	1.22 × 10^6^	1.45 × 10^6^
PD (a.u.)	1.18	1.18	1.23	1.03	1.18
*R*_a_ (μm)	3.03 ± 0.41	3.75 ± 0.24	3.93 ± 0.43	3.17 ± 0.46	2.44 ± 0.65
*R*_Z_DIN (μm)	16.5 ± 1.10	23.3 ± 1.83	24.1 ± 3.77	18.5 ± 3.71	15.4 ± 3.79
contact angle (deg)	75.3 ± 2.11	77.2 ± 1.39	79.1 ± 0.09	76.4 ± 1.49	74.2 ± 1.07
handling properties	brittle	flexible	brittle	flexible	flexible
Young’s modulus (MPa)	N/A	263 ± 35	N/A	54.5 ± 7.0	294 ± 38
tensile strength (MPa)	N/A	3.09 ± 0.18	N/A	1.06 ± 0.11	1.82± 0.19
strain at failure (%)	N/A	1.12 ± 0.03	N/A	1.00 ± 0.01	0.64 ± 0.02
conductivity (S cm^–1^)	2.24 × 10^–6^	3.66 × 10^–6^	3.46 × 10^–6^	3.07 × 10^–6^	1.36 × 10^–5^
granulation tissue depth at day 7 (mm)	3.0 ± 0.8	3.7 ± 0.6	3.8 ± 0.5	3.1 ± 0.5	5.1 ± 0.7
granulation tissue depth at day 28 (mm)	5.7 ± 2.8	6.9 ± 1.4	8.6 ± 1.2	6.1 ± 0.3	6.5 ± 0.7
granulation tissue depth at day 70 (mm)	8.3 ± 2.2	6.4 ± 0.9	10.7 ± 1.2	10.0 ± 1.9	12.5 ± 0.6

a Footnote: Mw (molecular weight), PD (polydispersity), *R*_a_ (average roughness, arithmetic average of the deviation from the mean line), R*_z_*DIN (average height difference between the five highest peaks and the five lowest valleys, determined in accordance with DIN 4768/1 as specified by the Deutsches Institut für Normung).

The EAPs were soluble in THF, and films were prepared by casting (optionally doped with camphorsulfonic acid, CSA [a simple inexpensive anionic dopant], or DMP [a clinically relevant anionic dopant] during solution processing) followed by vacuum drying, and characterization by a variety of techniques. The solution process yielded films with a thickness of ca. 50 μm and μm-scale roughness (*R*_a_ and R_Z_DIN) as determined by profilometry (Table 1), with similar water contact angles (Figure S19, Table 1).

Thermogravimetric analysis (TGA) of the films demonstrated the thermal stability of the polymers over the physiologically relevant temperature range, with initial mass loss ascribed to residual solvent/water in the films below 150 °C, and initial decomposition temperatures (IDTs) above 300 °C, and the low amounts of final residue confirm the degradation of these polymers at high temperatures (Figure S20, Table S1). Interestingly, the polymers incorporating hemin had higher IDTs (339–382 °C) than the polymers incorporating bilirubin or biliverdin (307 or 312 °C, respectively), and polymer **3** incorporating the PCL triol had the highest IDT.

The utility of materials in real-world applications is in part governed by their mechanical properties (i.e., robustness to handling/use), and while films of polymers **1** and **3** were brittle (correlating with their lower molecular weights, <4.2 × 10^5^ Da),^66^ films of polymers **2**, **4**, and **5** (all of which incorporated PCL diol 2000 and had molecular weights >1.2 × 10^6^ Da) were stable/flexible when manipulated by hand (Figure S21). The mechanical properties were assessed by tensile testing, and the Young’s moduli, tensile strength, and strain at failure are reported in Table 1 (Figures S22–S26); polymer **2** was observed to have the highest tensile strength of the three polymers, and the mechanical properties are analogous to those of a variety of soft human tissues.

UV–vis spectra of the EAP films (Figures S27 and S28) showed peaks characteristic of porphyrins (Soret band at ca. 400 nm and the weaker Q bands at ca. 500–700 nm). A similar increase in absorbance after doping with CSA was observed when polyaniline-ZnO nanocomposites were doped with CSA.^67^

Cyclic voltammetry (CV) was used to study the reduction/oxidation processes and electron transfer properties of the films of polymers **1–5** (Figure S29). Voltammograms of the materials show an anodic peak at ca. 0 V and the corresponding cathodic peak at ca. −0.6 V vs a Ag/AgCl reference electrode, and the somewhat unsymmetrical anodic/cathodic peaks are likely to be due to differences in background current and kinetic limitations.^68^ The conductance of the films was measured to be of the order of 10^–5^ to 10^–6^ S/cm, which is similar to analogous electroactive polymers.^29,41,69^

In silico, in vitro, and in vivo studies were used to assess the cytocompatibility of EAPs studied herein to understand the potential of such structures for eventual translation to real-world applications. In silico methods are being developed to facilitate polymer design, synthesis, processing, and characterization.^70^ Indeed, in silico toxicity screening methods have been developed to aid the design of bioactive molecules and minimize preclinical testing in vivo in animal models. We conducted in silico toxicity screening of the polymers and constituent building blocks (Table S2) using commercially available software, Derek Nexus, which identifies structural alerts for several endpoints;^71^ Sarah Nexus, a statistical-based model focused on mutagenicity only;^72^ and Zeneth, an expert, knowledge-based software that delivers accurate forced degradation predictions (Derek Nexus: v. 6.0.1, Nexus: 2.2.2; Sarah Nexus: v. 3.0.0, Sarah Model: 2.0; Zeneth: v. 8.1.1) that we have previously employed during the development of biomaterials.^38,73^ In silico toxicity screening highlighted the fact that the polymers all contain conjugated alkenes that have been associated with hepatotoxicity with several animal studies detailing the ability of these groups to change the liver enzyme levels causing necrosis, steatosis, and hepatocellular hypertrophy.^74^ The presence of aryl propionic acid groups has been shown to cause hepatotoxicity and mitochondrial dysfunction in mammals; arylacetic and arylpropionic acid groups are widely found as anti-inflammatory drugs; however, they are associated with hepatotoxicity similar to acute/chronic hepatitis^75^ and these toxic effects are rarely observed, with mild elevations in serum transaminases being the most common occurrence; in more serious cases, severe hepatocellular and cholestatic injuries may be observed, but these cases are very rare.^76^ Polymer **3** incorporating the PCL triol derivative was highlighted as containing similar functional groups to that of a 1,3-propanediol derivative, which has been associated with nephrotoxicity in mammals, which is a clear concern. Interestingly, the forced degradation predictions produced by Zeneth identified ester hydrolysis as the most likely degradation pathway, resulting in the generation of oligomers of block copolymers, starting materials, and oligomeric degradation byproducts of the PCL blocks, which should present minimal issues if the materials based on polymers **1–5** are implanted.

**Figure 2:**
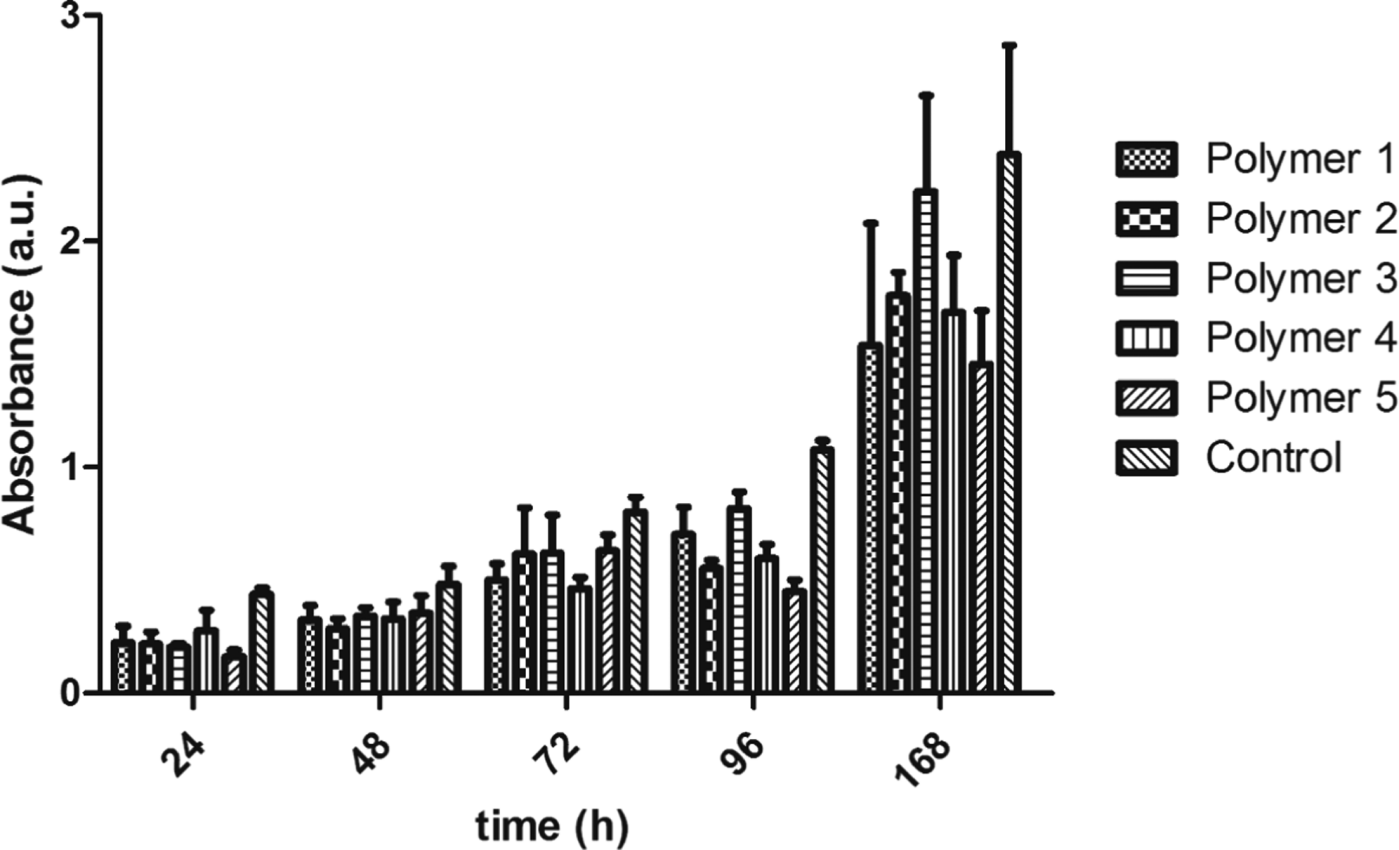
Cell adhesion for fibroblasts on films of polymers 1–5.

In vitro studies were conducted to assess the adhesion and proliferation of NIH3T3 fibroblasts on the surfaces of the polymer films. Confocal microscopy and the standard 3-(4,5-dimethylthiazol-2-yl)-2,5-diphenyltetrazolium bromide (MTT) assay^77−79^ were used to study cell adhesion and proliferation over the period of 1 week compared to the cells on tissue culture plastic, assessed at 1, 2, 3, 4, and 7 days (Figures 2 and S30, Table S3). NIH3T3 fibroblasts on polymers **1**–**5** adhered and proliferated over 4 days, albeit slightly less effectively than on tissue culture plastic; it is noteworthy that the fibroblasts proliferated for 7 days on all five polymers with a consistent rate of growth, although there were notable differences in adhesion relative to the tissue culture plastic controls. Interestingly, there was a correlation between the surface roughness of the films (Ra and RzDIN), water contact angle, and cell adhesion, with less adhesion to the polymer **5** films (smoother and more hydrophilic) than the polymer **3** films (rougher and more hydrophobic), as the rougher and more hydrophobic films offer higher surface areas for adsorption of cell adhesive species (e.g., proteins like collagen-1 and/or laminin-1) to which the cells to adhere to.^80^ Nevertheless, the fact the films of polymers **1** and **3** are brittle makes the films of polymers **2**, **4**, and **5** more straightforward to handle/use in real life, and cells adhered to all of those films.

In vivo studies were conducted by subcutaneous implantation of the films in mice. Simple and effective sterilization methodologies for materials based on these polymers are important for their potential future use. Autoclaving with dry heat or high-pressure saturated steam was ruled out due to the melting and/or hydrolysis of the polymers. The polymer films were stable to brief exposure to aqueous ethanol followed by UV irradiation, which was the pragmatic solution for in vivo studies in this report; however, sterilization by exposure to ethylene oxide (EO), gamma irradiation, or electron beam is more likely to be the methodology in studies bringing such materials to higher technology readiness levels.^81,82^

**Figure 3:**
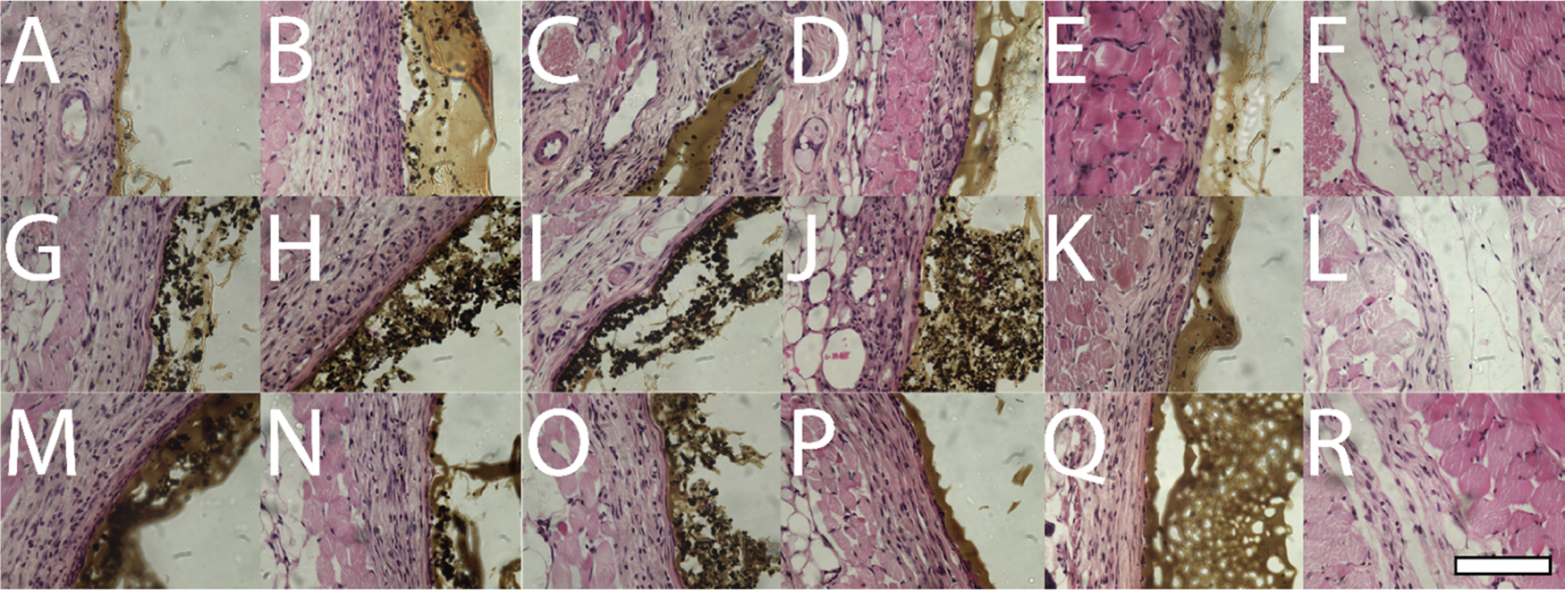
Representative images of the area of tissue adjacent to the subcutaneous implants in Balb/C mice cultured on day 7 (A–F), day 28 (G–L), or day 70 (M–R). Samples stained with Harris’s hematoxylin and eosin (A, G, M), polymer 1 (B, H, N), polymer 2 (C, I, and O), polymer 3 (D, J, and P), polymer 4 (E, K, and Q), polymer 5 (F, L, and R). Sham surgery control—no film implanted. Scale bar represents 100 μm.

No signs of deleterious effects were seen during the study; there were no unexpected fluctuations in bodyweight (Figure S31), nor was there evidence of inflammation surrounding the implants upon gross examination at the time of euthanization (Figure S32). Histological examination of the area adjacent to the materials showed the presence of an area of fibrous granulation tissue, with an increase in thickness from day 7 to day 28 and day 70 as would be expected (representative images are shown in Figure 3). The mean thickness of the granulation tissue in mm is shown in Table 1. Although some outliers were seen, with films of polymer **5** eliciting a greater thickness on day 7, and day 70, it was within range on day 28, and the granulation tissue for polymer **2** on day 70 was thinner than the other samples, there was nothing consistent throughout the samples to suggest any significant lack of biocompatibility for any of the biomaterials tested. This was supported by differential blood count data where values were mostly within normal expected ranges (Table S4), and it is noteworthy that this is an analogous outcome to nondegradable electroactive polymers (e.g., polyaniline, polypyrrole, and PEDOT) implanted in vivo, which are comparable to FDA-approved poly(lactic-*co*-glycolic acid).^34^

**Figure 4:**
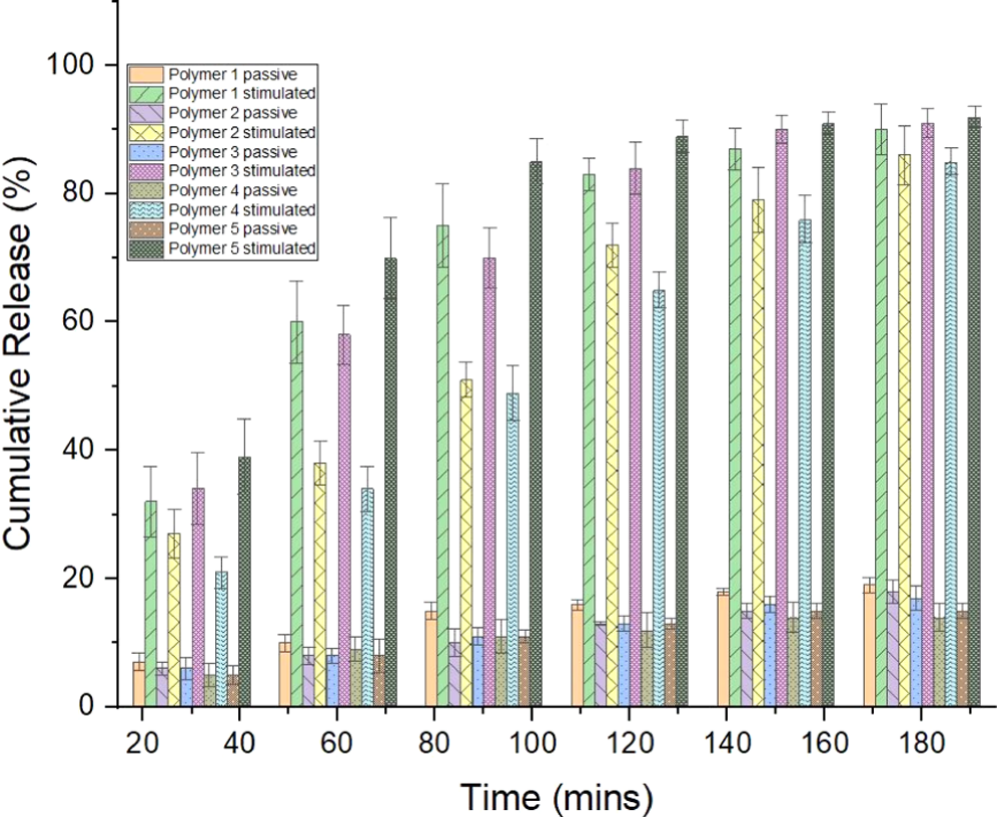
DMP release study from films of polymers 1–5. Measurements every 30 min from samples without stimulation (i.e., passive release) or with electrical stimulation.

In vitro drug delivery studies were conducted using DMP (Figure S33), a steroidal anti-inflammatory derived from dexamethasone, a corticosteroid hormone, that has been widely used in the treatment of a variety of conditions such as allergies, arthritis, respiratory problems, cancers, and skin diseases.^60^ We studied the release of DMP from DMP-doped EAP films into phosphate-buffered saline (PBS) in the absence/presence of electrical stimuli via UV spectroscopy (Figure 4) over a period of 3 h (where electrical stimulation was applied, this was for 1 min every 15 min for the duration of the experiment). Passive release of DMP was observed from all DMP-doped films of polymers **1**–**5**; however, this amounted to less than 20% over the course of the experiment for all films; by comparison, the application of an electrical stimulus triggered the delivery of DMP from the films, with an increase of ca. 10–30% relative to the passive release control experiment for the specific polymer films. At the early stages of the release study, stimulated release was greater from DMP-doped films of polymers **1**, **3**, and **5** than the more mechanically robust films of polymers **2** and **4**; however, it was possible to release 80–90% of the drug from all of the polymer films as shown by cumulative release data at 180 min. Prior to reaching 85% of drug release, stimulation of the polymer film resulted in between 10 and 20% of release of the loaded DMP, but this dropped to 3–4% after 85% release. This degree of temporal control is particularly useful in cases where it is important to control the chronopharmacology of the drug in line with the chronobiology of the condition being treated,^3,83−85^ and we foresee opportunities for the development of such smart drug delivery systems for patient-specific treatments, offering significant beneficial economic, health, and societal impacts in the foreseeable future.

## Conclusions

Electroactive copolymers composed of blocks of polycaprolactone (PCL) and biorenewable electroactive pyrrole oligomers (bilirubin, biliverdin, and hemin) were prepared in a single step from commercially available starting materials. The polyesters were solution-processable, and their mechanical properties could be tuned based on the constituent building blocks. In vitro cell culture studies demonstrated NIH3T3 fibroblasts adhered and proliferated on the surfaces of the films (and that this correlated with surface roughness); in silico toxicity screening studies highlighted potentially adverse biological reactions to the polymers (albeit unlikely), and implantation of the films in vivo showed the films to be cytocompatible with no obvious inflammation relative to sham surgeries. DMP-doped films released a small amount of DMP passively; however, the application of an electrical stimulus was observed to markedly enhance this in vitro. Such cytocompatible stimuli-responsive biomaterials have significant potential for the delivery of bioactive molecules in agriculture, and human/veterinary medicine, particularly the flexible materials based on polymers **2**, **4**, and **5**.

## Supporting Information Available

Chemical structures of materials studied and synthetic scheme; IR spectra; NMR spectra; GPC data; photographs of representative contact angle measurements; TGA data; mechanical property data; UV–vis spectra; voltammograms; in silico toxicity prediction data; in vitro cell culture data; and in vivo implantation data (PDF)

## Notes

In vivo studies were conducted in accordance with ethical standards approved by the Animal Welfare Ethics Review Board at the University of Bradford and in accordance with the UK National Cancer Research Institute Guidelines for the Welfare of Animals.^86^ Throughout the study, all of the mice were housed in air-conditioned rooms in facilities approved by the United Kingdom Home Office to meet all current regulations and standards. All procedures were carried out under a Project Licence (PB7ACB920) issued by the UK Home Office according to government legislation.
