# Correction to “Controlled Bioactive Delivery Using Degradable Electroactive Polymers”

**DOI:** 10.1021/acs.biomac.5c00295

**Published:** 2025-09-15

**Authors:** Mark D. Ashton, Patricia A. Cooper, Sofia Municoy, Martin F. Desimone, David Cheneler, Steven D. Shnyder, John G. Hardy

We found an error in the original manuscript in the experimental section describing the drug delivery studies in vitro, where the amount of time the cell was allowed to rest before spectroscopy should be 29 min, likewise in the results and discussion (where electrical stimulation was applied, this was for 1 min every 30 min for the duration of the experiment). The findings in the paper are unchanged by the Correction.

We found an error in the Supporting Information in the original manuscript with Figure S7 and S8 being identical (copied and pasted from a PhD thesis Supporting Information document); we have corrected this. Additionally, we have replotted Figures S1–S11 to ensure consistency of formatting. The findings in the paper are unchanged by the Correction.

## Supplementary Material



## Notes

Corrected Figures S1–S11 (PDF)

