# Supplementary material for: Controlled Bioactive Delivery Using Degradable Electroactive Polymers
Source: Biomacromolecules. 2022 Jun 24;23(7):3031–40. doi: 10.1021/acs.biomac.2c00516 (PMC9277582; doi:10.1021/acs.biomac.2c00516)
Supplement: Supplementary file 1 — bm2c00516_si_001.pdf [file bm2c00516_si_001.pdf]

Controlled bioactive delivery using degradable electroactive polymers

Mark D. Ashton,[a] Patricia A. Cooper,[b] Sofia Municoy,[c] Martin F. Desimone,[c] David Cheneler[d,e], Steven D. Shnyder,[b] and John G. Hardy[a,e,*]

a Department of Chemistry, Faculty of Science and Technology, Lancaster University, Bailrigg, Lancaster, LA1 4YB, UK.

b Institute of Cancer Therapeutics, School of Pharmacy and Medical Sciences, Faculty of Life Sciences, University of Bradford, Bradford, BD7 1DP, UK.

c Instituto de Química y Metabolismo del Fármaco (IQUIMEFA), Facultad de Farmacia y Bioquímica, Consejo Nacional de Investigaciones, Científicas y Técnicas (CONICET), Universidad de Buenos Aires, Junín 956, Piso 3° (1113), Buenos Aires 1113, Argentina.

d Department of Engineering, Faculty of Science and Technology, Lancaster University, Bailrigg, Lancaster, LA1 4YW, UK.

e Materials Science Institute, Lancaster University, Bailrigg, Lancaster, LA1 4YB, UK.

Supporting Information

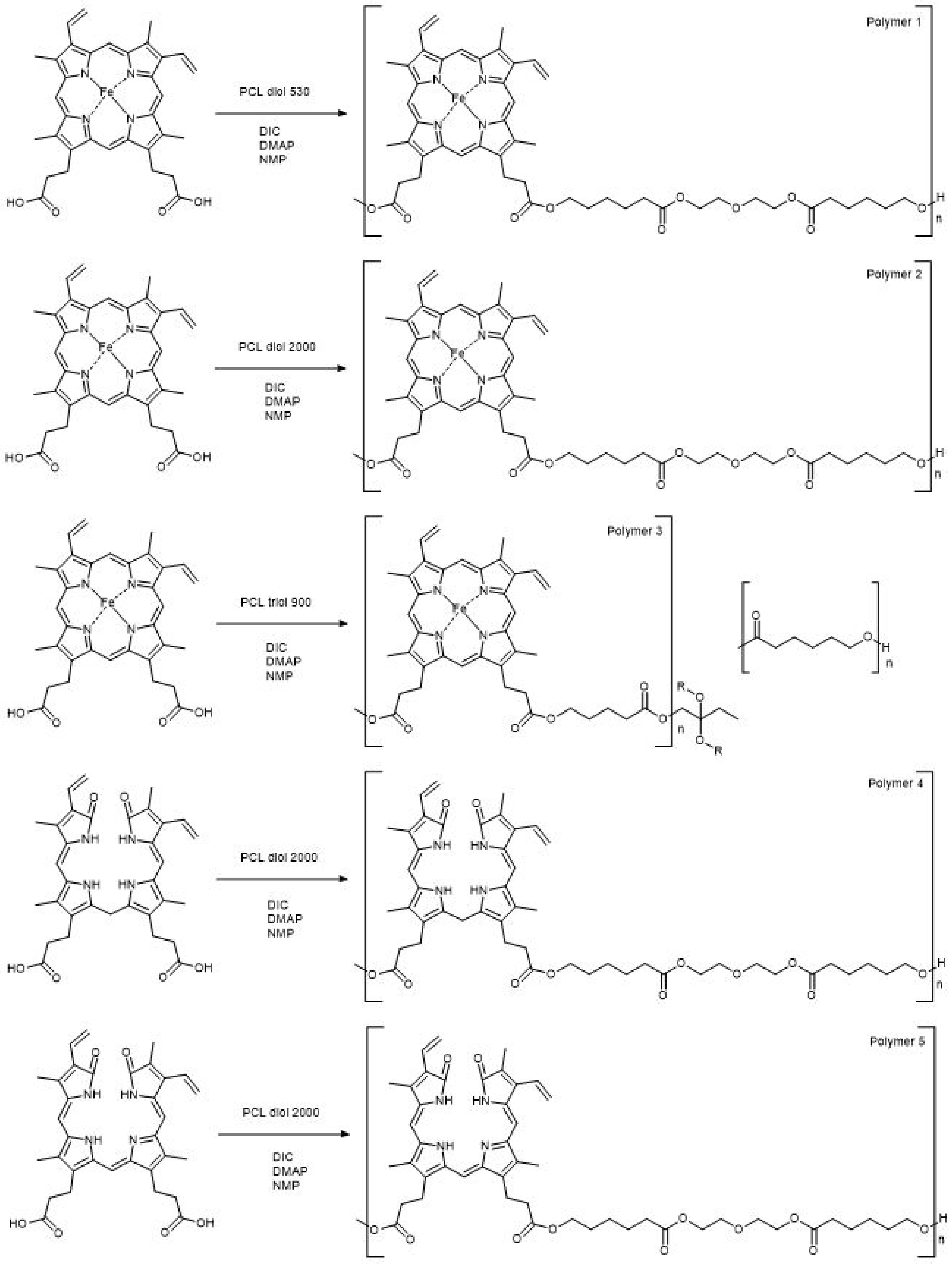

Scheme S1. Synthesis of EAPs via the Steglich esterification employing DIC (Diisopropylcarboiimide), DMAP (4-(Dimethylamino)pyridine) and NMP (N-Methyl-2-pyrrolidone). Polymers 1-3 incorporating hemin, polymer 4 incorporating bilirubin, and polymer 5 incorporating biliverdin.

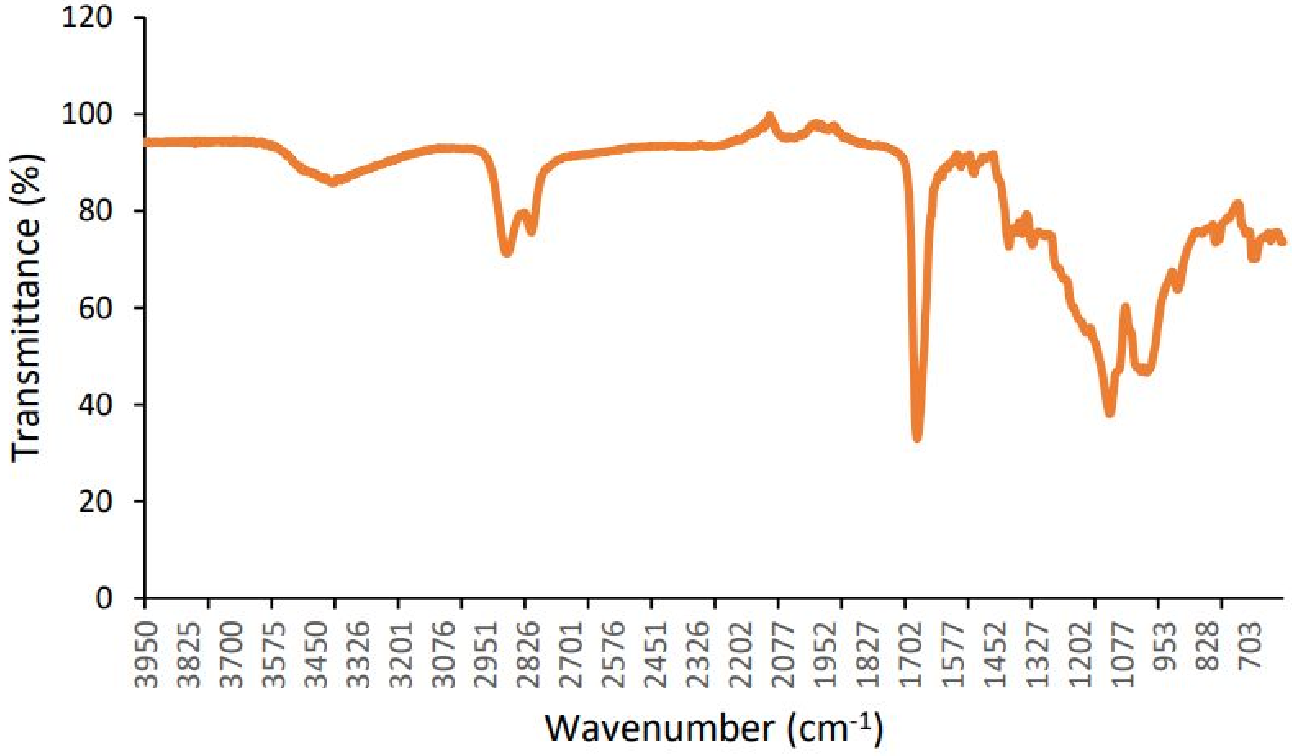

Figure S1. IR spectrum of alcohol-terminated PCL Diol 2000 g.mol^{-1}.

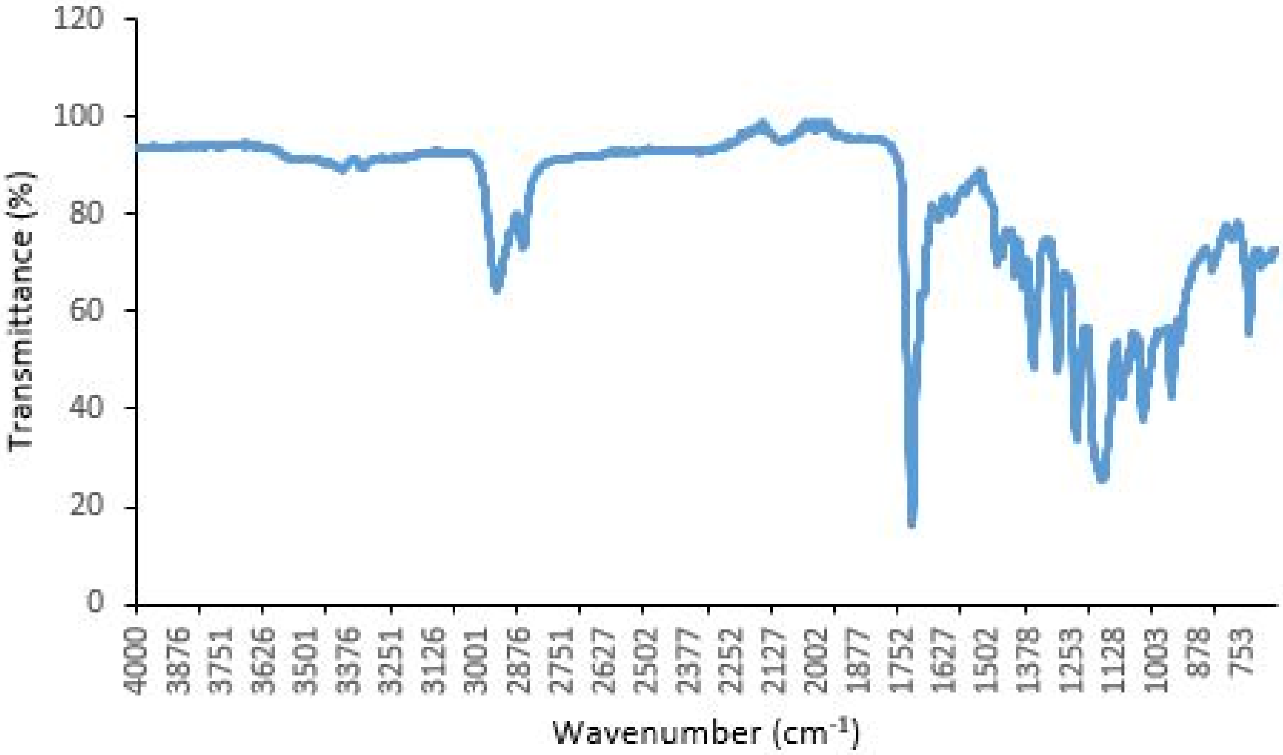

Figure S2. IR spectrum of PCL Diol 530 g.mol^{-1}.

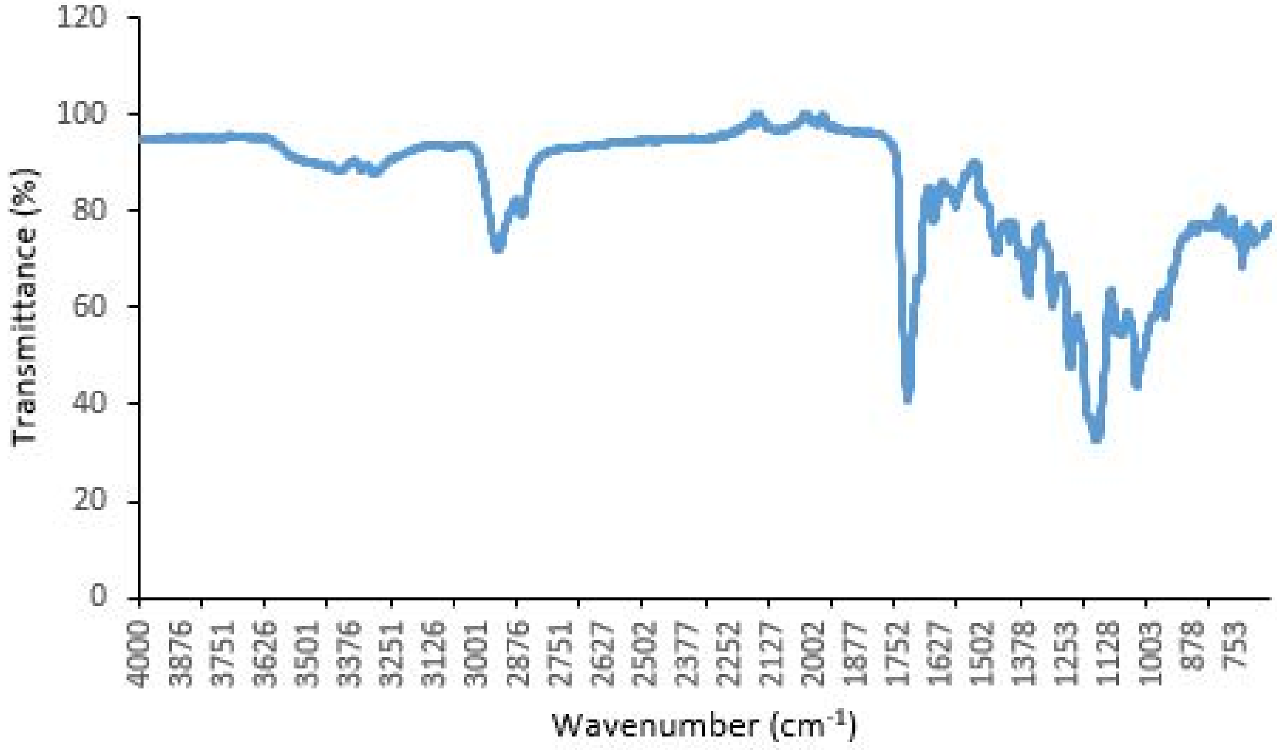

Figure S3. IR spectrum of PCL Triol 900 g.mol^{-1}.

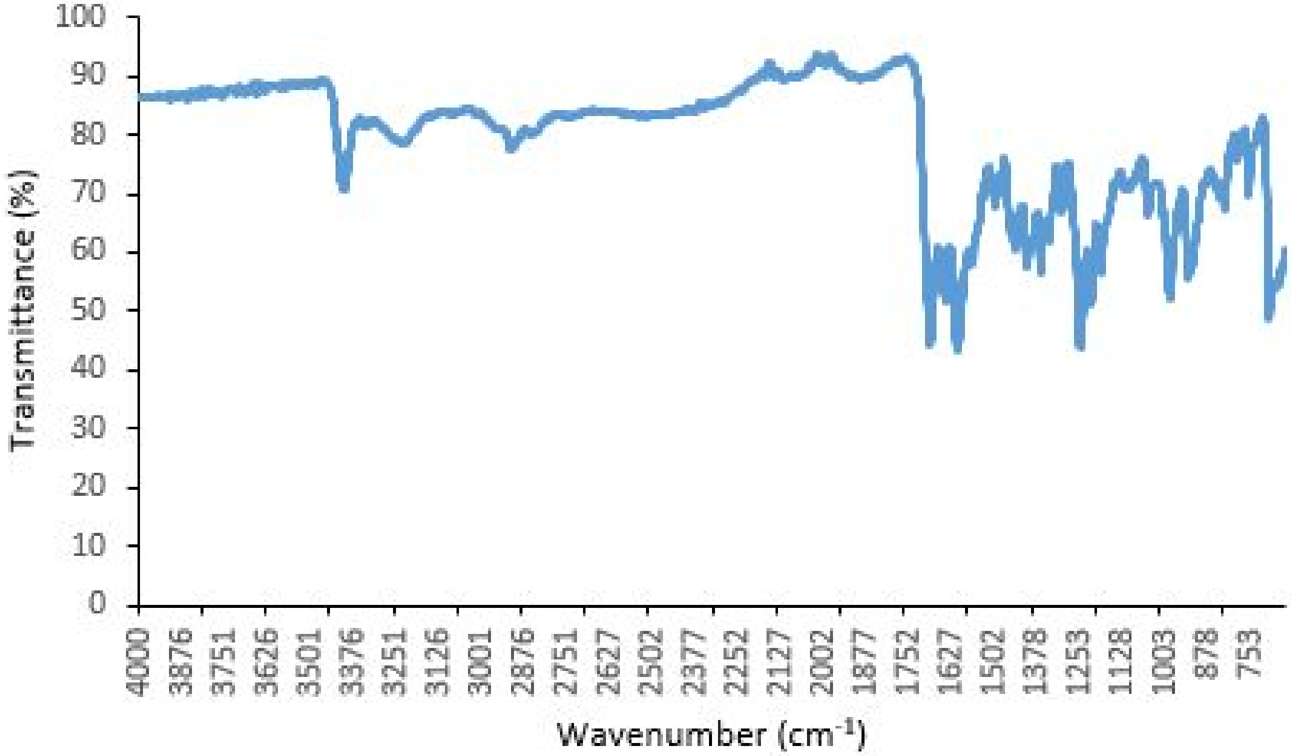

Figure S4. IR spectrum of Bilirubin.

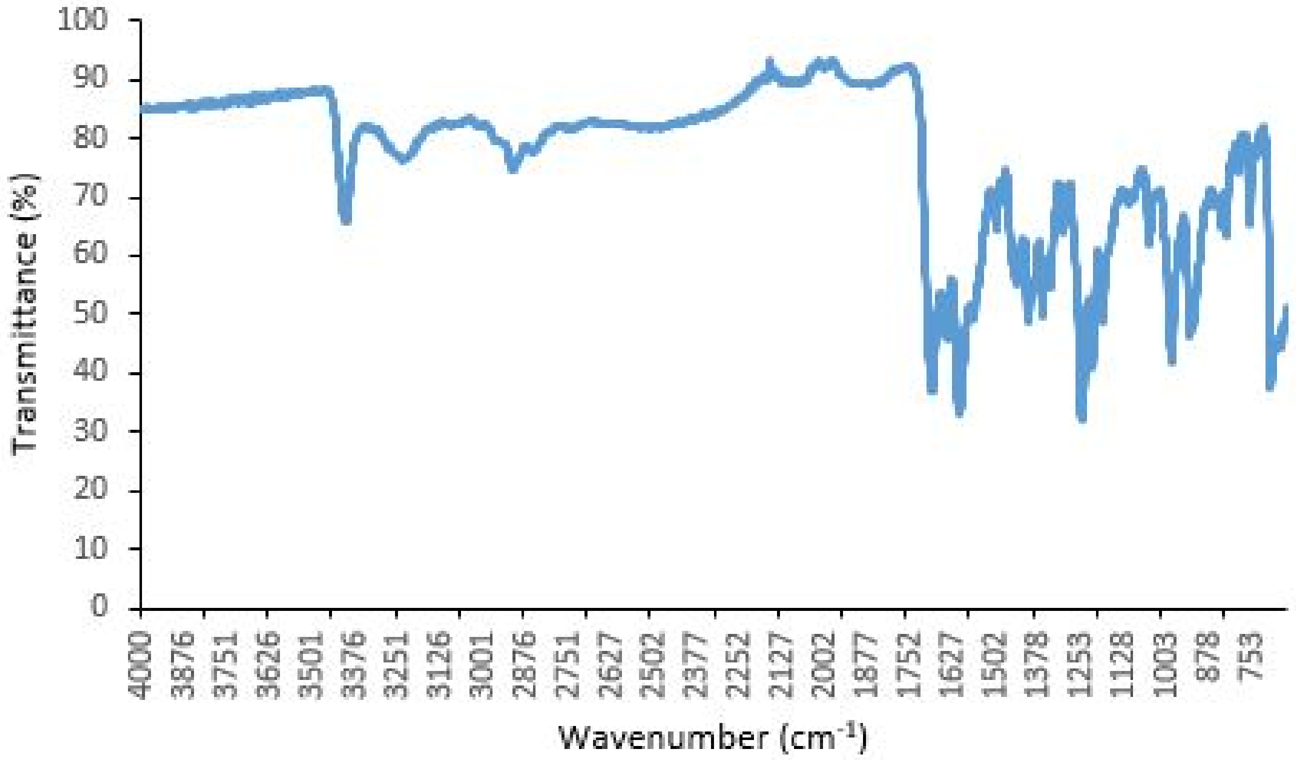

Figure S5. IR spectrum of Biliverdin.

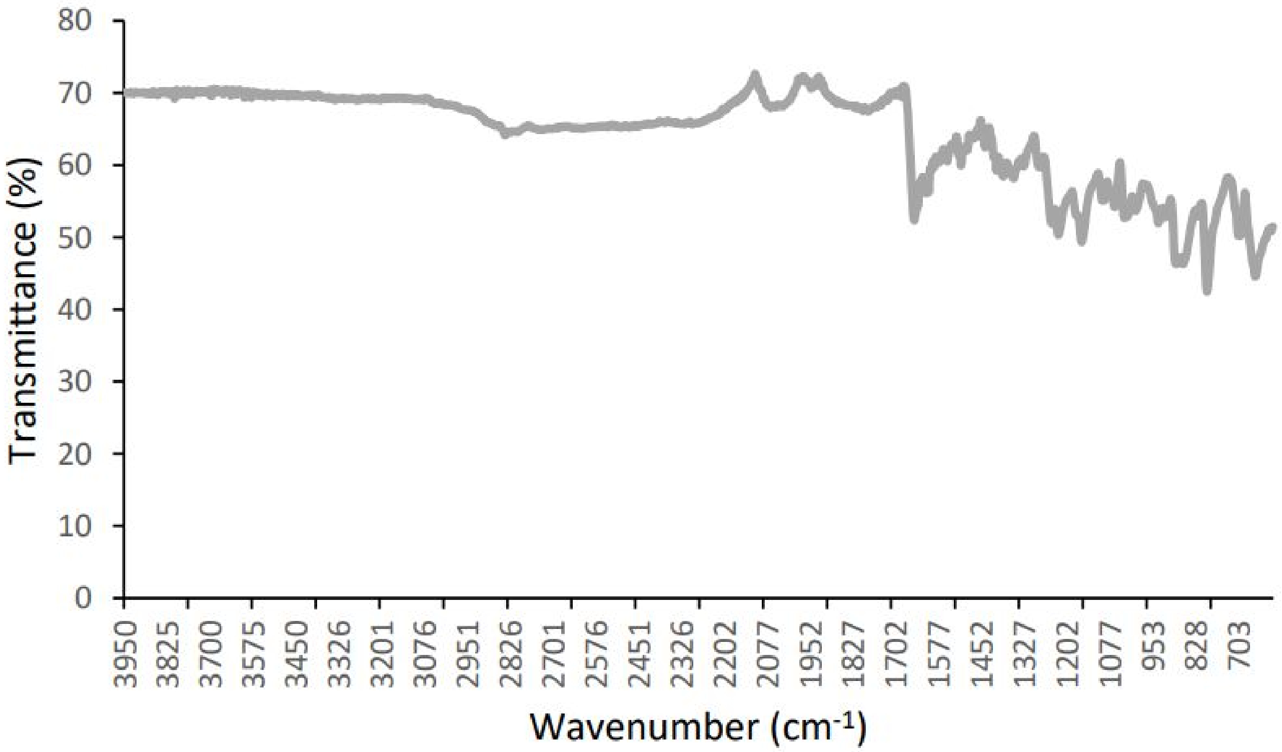

Figure S6. IR spectrum of hemin.

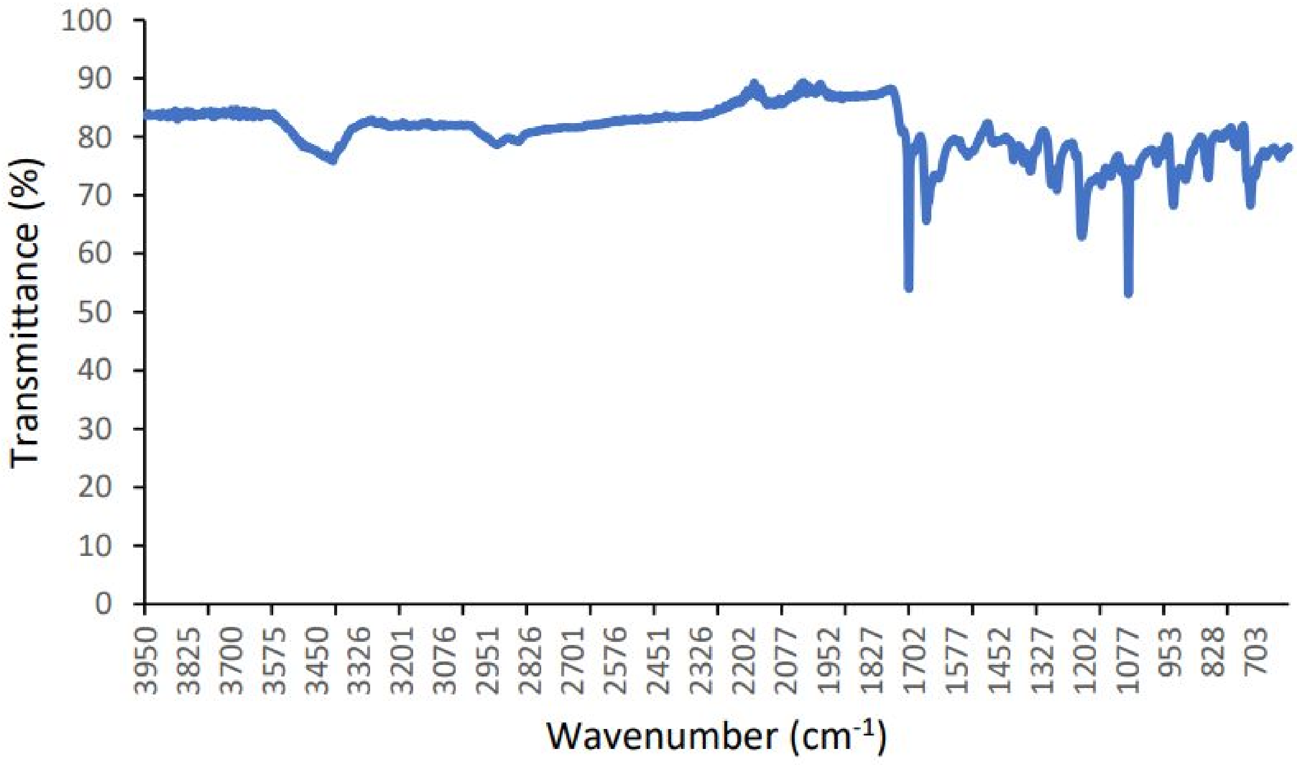

Figure S7. IR spectrum of polymer 1.

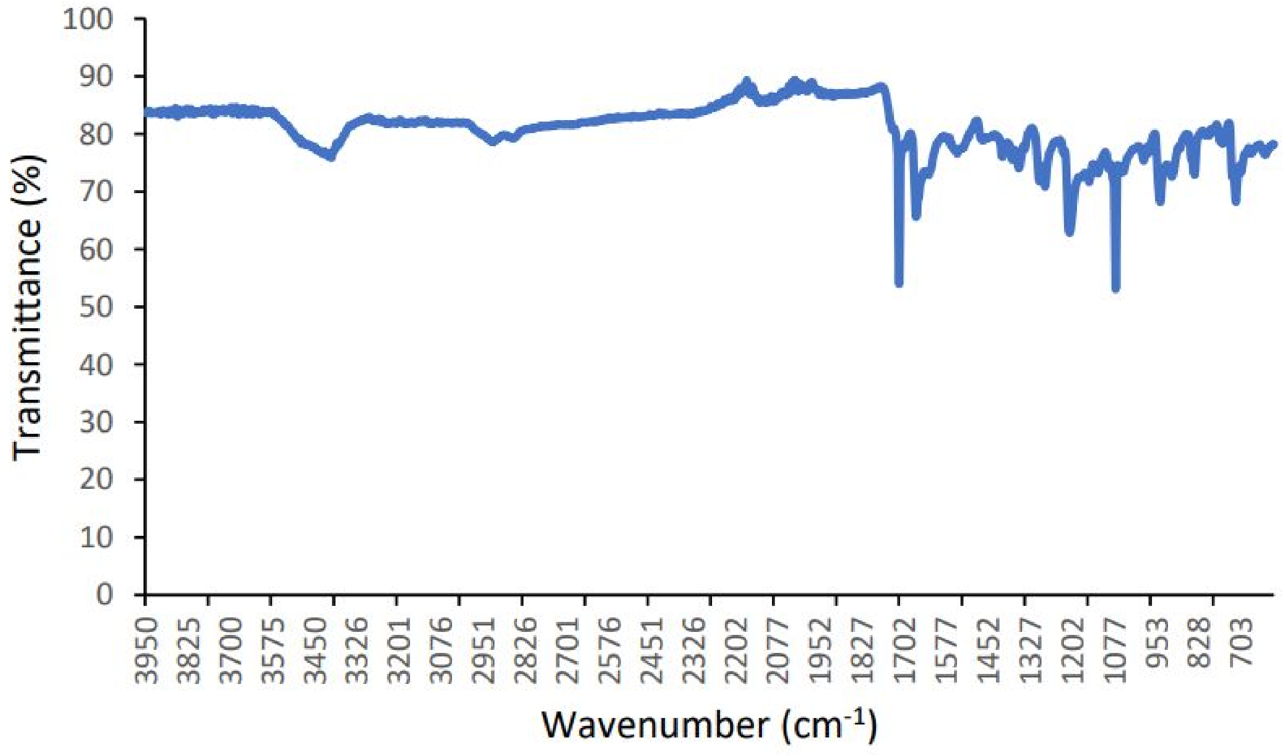

Figure S8. IR spectrum of polymer 2.

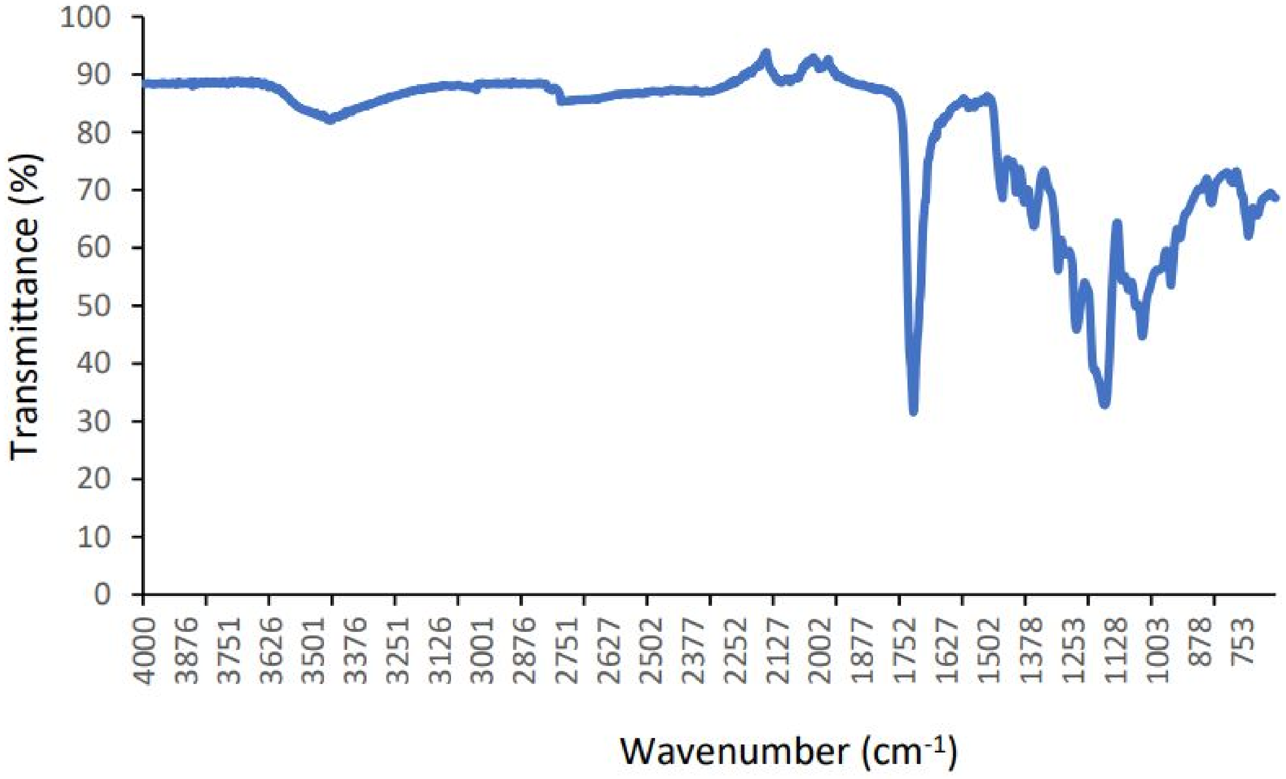

Figure S9. IR spectrum of polymer 3.

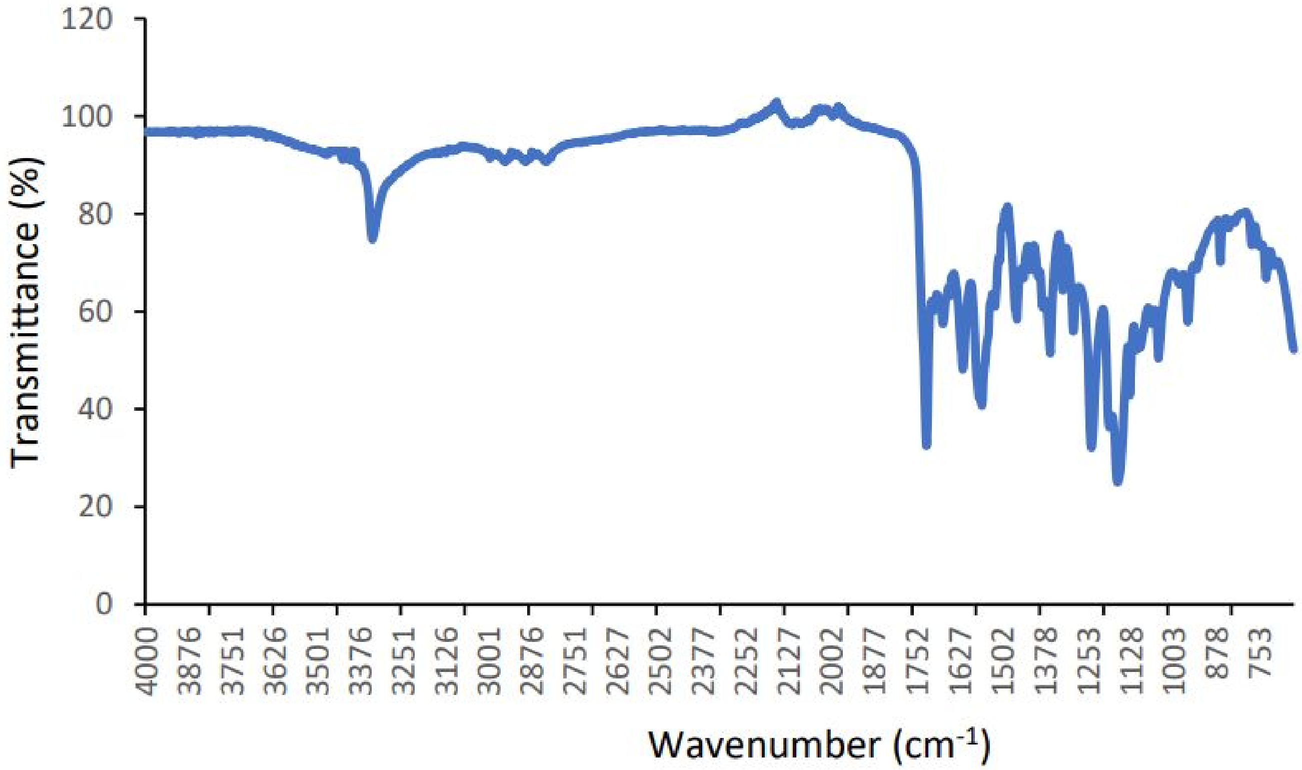

Figure S10. IR spectrum of polymer 4.

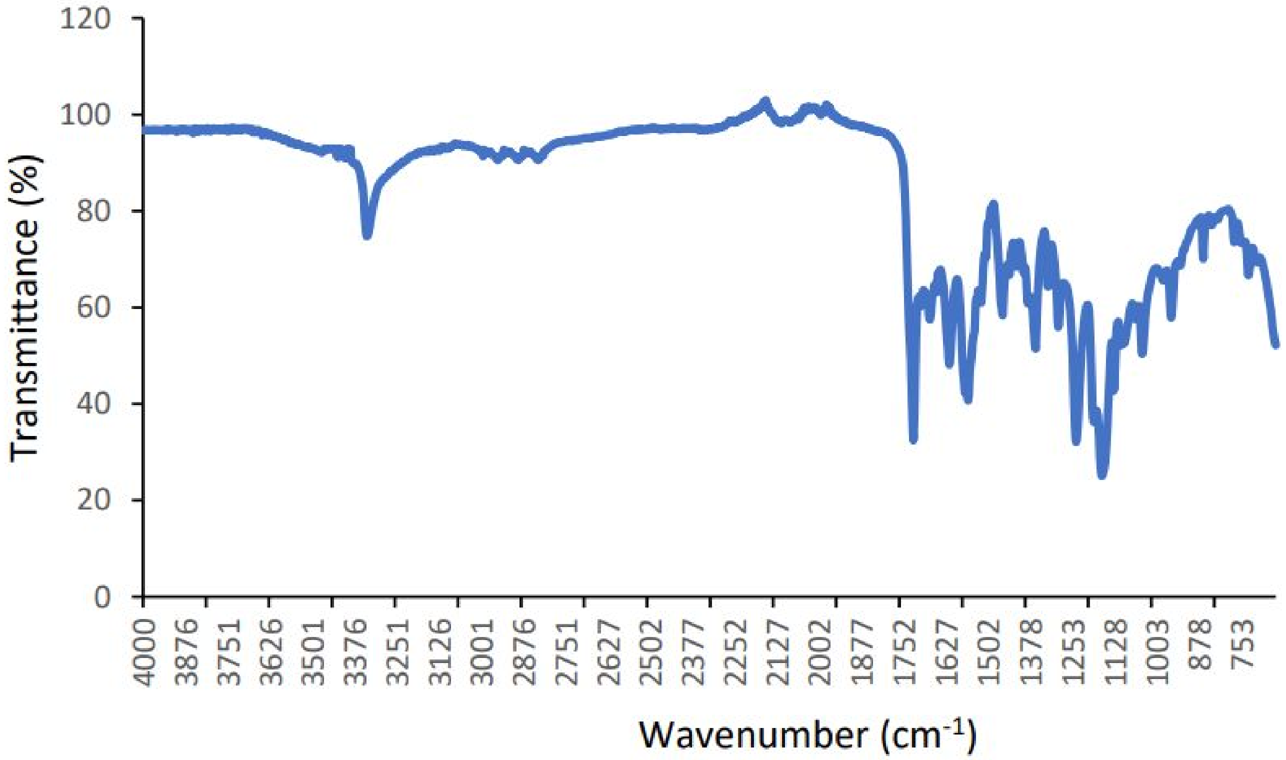

Figure S11. IR spectrum of polymer 5.

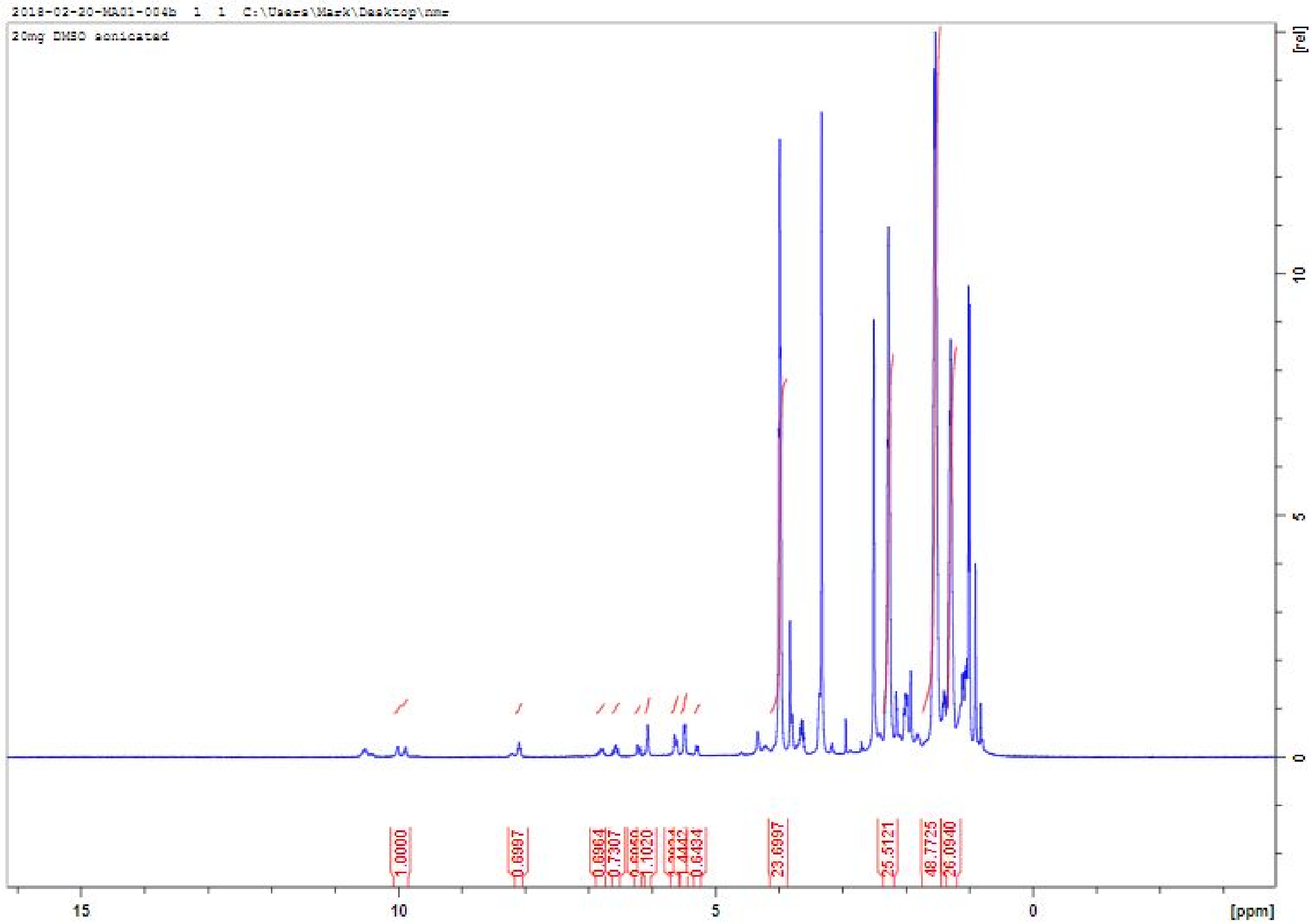

Figure S12. NMR spectrum of polymer 4.

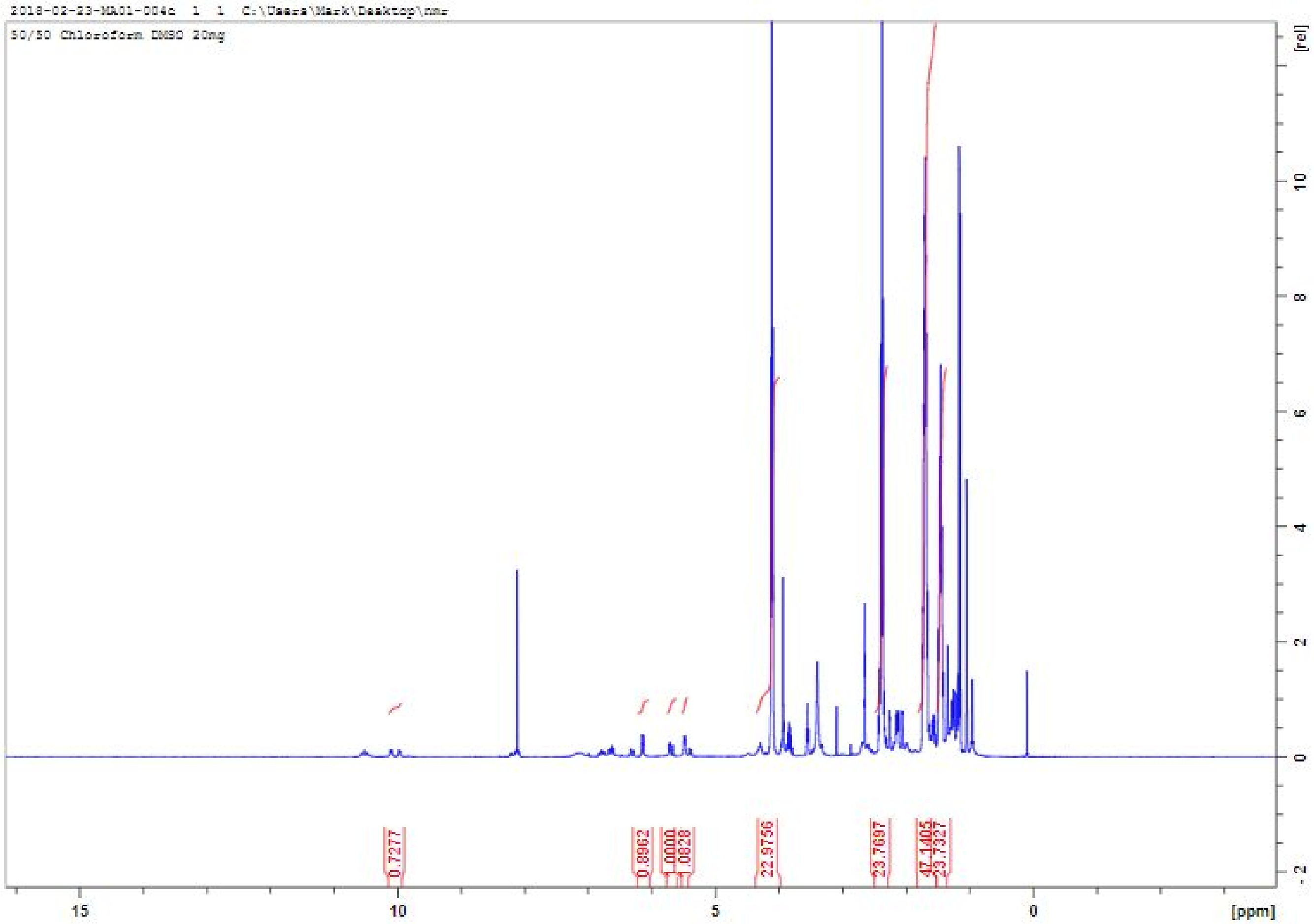

Figure S13. NMR spectrum of polymer 5.

ASTRA 6 Report MA1[Sequence1]

Peak Results

	Peak 1
**Hydrodynamic radius (v) moments (nm)**	
Rh(v)n	6.738 (±7.199%)
Rh(v)w	7.093 (±8.089%)
Rh(v)z	7.435 (±8.820%)
**Masses**	
Calculated Mass (µg)	37.44
Mass Recovery (%)	n/a
Mass Fraction (%)	100.0
**Molar mass moments (g/mol)**	
Mn	3.539×10^{5} (±19.986%)
Mp	6.462×10^{5} (±31.118%)
Mv	3.816×10^{5} (±3.588%)
Mw	4.164×10^{5} (±25.374%)
Mz	4.776×10^{5} (±60.085%)
**Polydispersity**	
Mw/Mn	1.177 (±32.300%)
Mz/Mn	1.350 (±63.322%)
**rms radius moments (nm)**	
Rn	98.7 (±14.8%)
Rw	98.1 (±16.8%)
Rz	97.9 (±18.4%)
**Intrinsic viscosity moments (mL/g)**	
[η]n	5.794 (±2.629%)
[η]w	5.73 (±2.55%)
[η]z	5.679 (±2.467%)

Mark-Houwink-Sakurada a: -0.090 (±9.696%)
Mark-Houwink-Sakurada K: 1.848×10^{1} (±11.280%) mL/g

Figure S14. GPC data for polymer 1.

ASTRA 6 Report MA5[Sequence1]

Peak Results	Peak 1
Hydrodynamic radius (v) moments (nm)	
Rh(v)n	20.761 (±2.782%)
Rh(v)w	21.612 (±2.370%)
Rh(v)z	22.080 (±2.115%)
Masses	
Calculated Mass (μg)	1.70
Mass Recovery (%)	n/a
Mass Fraction (%)	100.0
Molar mass moments (g/mol)	
Mn	1.226×10^{6} (±5.651%)
Mp	1.947×10^{6} (±3.198%)
Mv	1.299×10^{6} (±2.159%)
Mw	1.449×10^{6} (±4.300%)
Mz	1.596×10^{6} (±9.091%)
Polydispersity	
Mw/Mn	1.182 (±7.102%)
Mz/Mn	1.301 (±10.705%)
rms radius moments (nm)	
Rn	n/a
Rw	n/a
Rz	n/a
Intrinsic viscosity moments (mL/g)	
[η]n	50.752 (±7.072%)
[η]w	46.98 (±5.91%)
[η]z	44.331 (±5.099%)

Mark-Houwink-Sakurada a: -0.443 (±5.200%)

Mark-Houwink-Sakurada K: 2.427×10^{4} (±33.396%) mL/g

Figure S15. GPC data for polymer 2.

ASTRA 6 Report MA3[Sequence1]

Peak Results

	Peak 1
**Hydrodynamic radius (v) moments (nm)**	
Rh(v)n	4.584 (±16.542%)
Rh(v)w	5.233 (±16.963%)
Rh(v)z	5.698 (±17.464%)
**Masses**	
Calculated Mass (μg)	0.82
Mass Recovery (%)	n/a
Mass Fraction (%)	100.0
**Molar mass moments (g/mol)**	
Mn	1.840×10^{4} (±40.256%)
Mp	6.794×10^{3} (±53.235%)
Mv	2.189×10^{4} (±9.094%)
Mw	2.259×10^{4} (±47.954%)
Mz	2.550×10^{4} (±110.703%)
**Polydispersity**	
Mw/Mn	1.227 (±62.611%)
Mz/Mn	1.386 (±117.795%)
**rms radius moments (nm)**	
Rn	n/a
Rw	n/a
Rz	n/a
**Intrinsic viscosity moments (mL/g)**	
[η]n	37.412 (±16.531%)
[η]w	43.14 (±16.69%)
[η]z	47.681 (±16.758%)

Mark-Houwink-Sakurada a: 0.600 (±10.306%)

Mark-Houwink-Sakurada K: 1.105×10^{-1} (±65.776%) mL/g

Figure S16. GPC data for polymer 3.

ASTRA 6 Report MA4[Sequence1]

Peak Results

	Peak 1
**Hydrodynamic radius (v) moments (nm)**	
Rh(v)n	16.555 (±1.321%)
Rh(v)w	16.444 (±1.380%)
Rh(v)z	16.331 (±1.450%)
**Masses**	
Calculated Mass (μg)	14.54
Mass Recovery (%)	n/a
Mass Fraction (%)	100.0
**Molar mass moments (g/mol)**	
Mn	1.183×10^{6} (±0.493%)
Mp	1.122×10^{6} (±0.297%)
Mv	1.173×10^{6} (±0.100%)
Mw	1.222×10^{6} (±0.447%)
Mz	1.261×10^{6} (±0.969%)
**Polydispersity**	
Mw/Mn	1.032 (±0.665%)
Mz/Mn	1.065 (±1.087%)
**rms radius moments (nm)**	
Rn	n/a
Rw	n/a
Rz	n/a
**Intrinsic viscosity moments (mL/g)**	
[η]n	25.542 (±3.743%)
[η]w	24.32 (±3.85%)
[η]z	23.099 (±4.006%)

Mark-Houwink-Sakurada a: -1.583 (±1.593%)

Mark-Houwink-Sakurada K: 9.784×10^{10} (±35.833%) mL/g

Figure S17. GPC data for polymer 4.

ASTRA 6 Report MA5[Sequence1]

Peak Results

	Peak 1
**Hydrodynamic radius (v) moments (nm)**	
Rh(v)n	20.761 (±2.782%)
Rh(v)w	21.612 (±2.370%)
Rh(v)z	22.080 (±2.115%)
**Masses**	
Calculated Mass (μg)	1.70
Mass Recovery (%)	n/a
Mass Fraction (%)	100.0
**Molar mass moments (g/mol)**	
Mn	1.226×10^{6} (±5.651%)
Mp	1.947×10^{6} (±3.198%)
Mv	1.299×10^{6} (±2.159%)
Mw	1.449×10^{6} (±4.300%)
Mz	1.596×10^{6} (±9.091%)
**Polydispersity**	
Mw/Mn	1.182 (±7.102%)
Mz/Mn	1.301 (±10.705%)
**rms radius moments (nm)**	
Rn	n/a
Rw	n/a
Rz	n/a
**Intrinsic viscosity moments (mL/g)**	
[η]n	50.752 (±7.072%)
[η]w	46.98 (±5.91%)
[η]z	44.331 (±5.099%)

Mark-Houwink-Sakurada a: -0.443 (±5.200%)

Mark-Houwink-Sakurada K: 2.427×10^{4} (±33.396%) mL/g

Figure S18. GPC data for polymer 5.

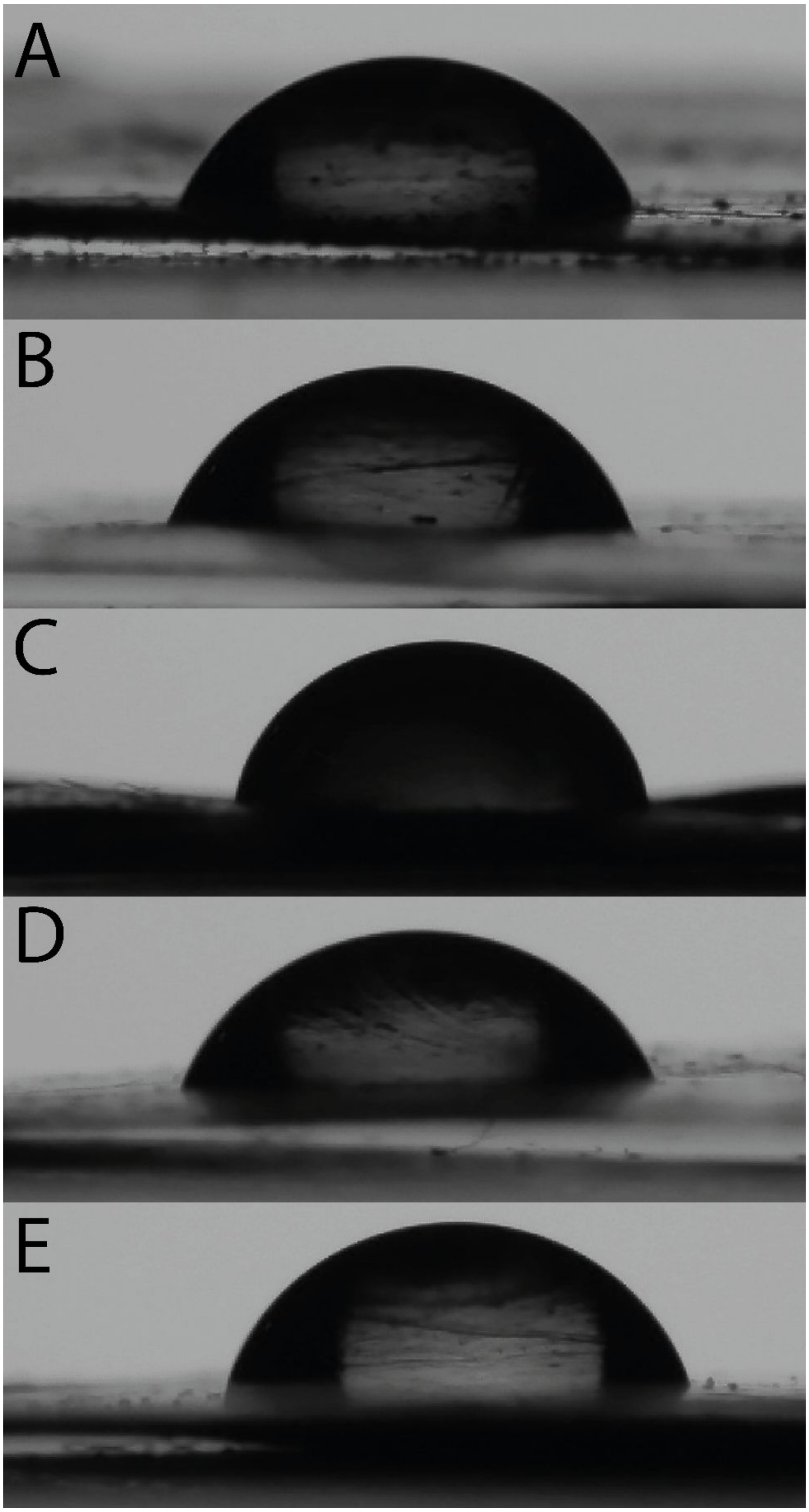

Figure S19. Representative images of contact angle measurements for polymer films. **A)** Polymer 1. **B)** Polymer 2. **C)** Polymer 3. **D)** Polymer 4. **E)** Polymer 5.

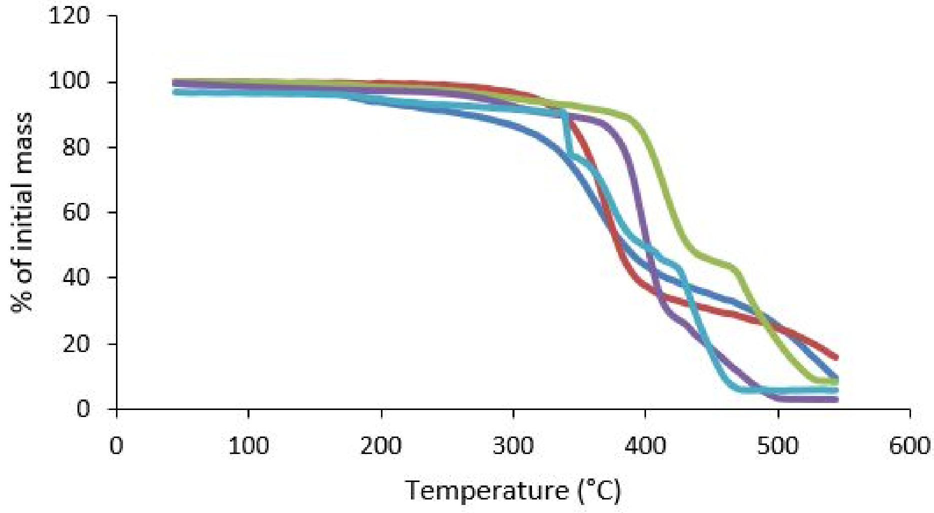

Figure S20. TGA curve for polymer 1 (light blue), polymer 2 (purple), polymer 3 (green), polymer 4 (red) and polymer 5 (dark blue) demonstrating the initial decomposition temperature and final residue.

Table S1. Properties of polymers **1-5**.

**Polymer**	**1** (PCL diol 530 + hemin)	**2** (PCL diol 2000 + hemin)	**3** (PCL triol 900 + hemin)	**4** (PCL diol 2000 + bilirubin)	**5** (PCL diol 2000 + biliverdin)
**IDT (°C)**	339	369	382	307	312
**$D_{1/2}$ (°C)**	416	413	462	369	358
**Final Residue (%)**	5.89	3.23	8.28	15.5	9.47

IDT: Initial decomposition temperature. $D_{1/2}$: Temperature at which 50% of the material has decomposed.

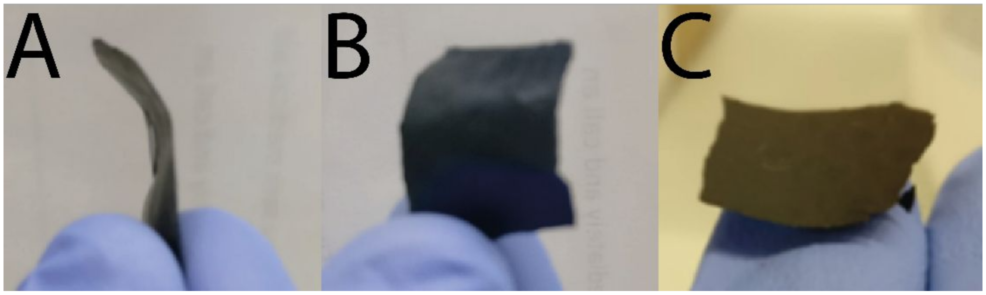

Figure S21. Photographs of polymers during manual handling. A) Polymer 2 (left). Polymer 4 (middle). C) Polymer 5 (right).

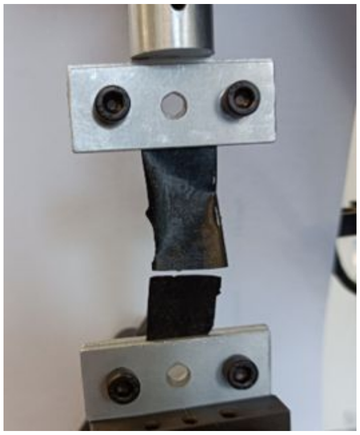

Figure S22. Experimental setup for acquisition of mechanical data.

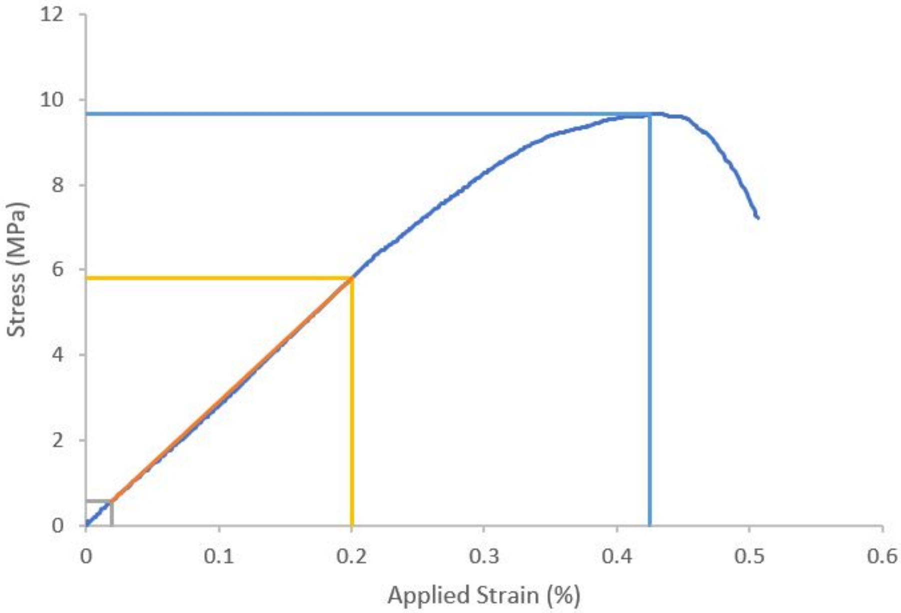

Figure S23. Example stress strain curve and points used for data extraction. Data points: Yellow – High strain limit, Blue – Failure point, Grey – Low strain limit, Orange – Tangent.

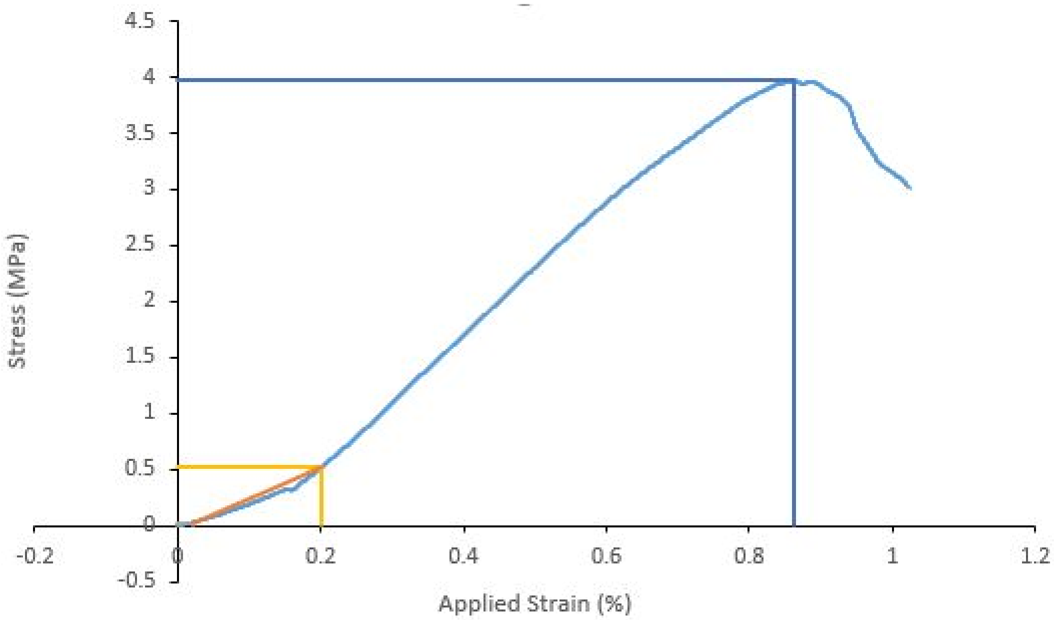

Figure S24. Representative stress strain curve for polymer 2.

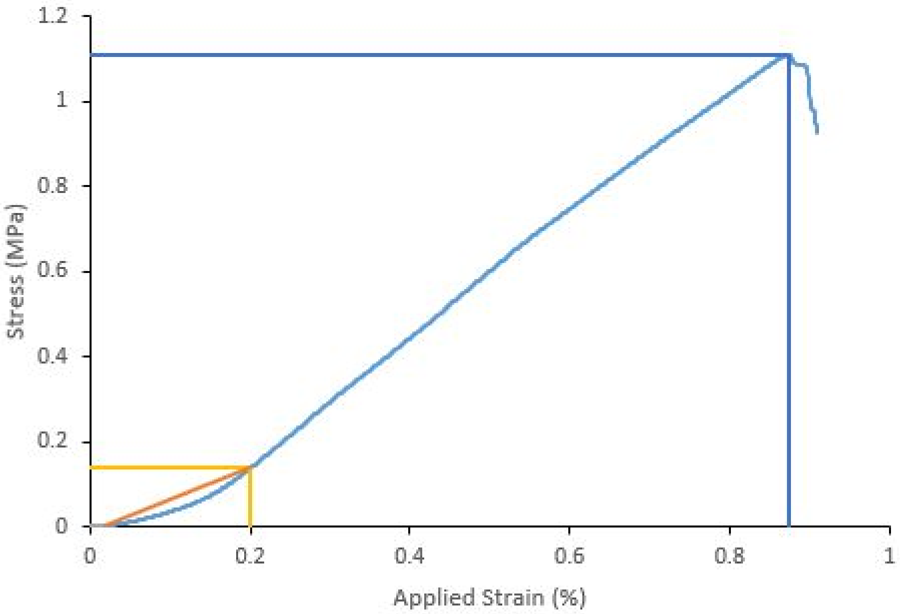

Figure S25. Representative stress strain curve for polymer 4.

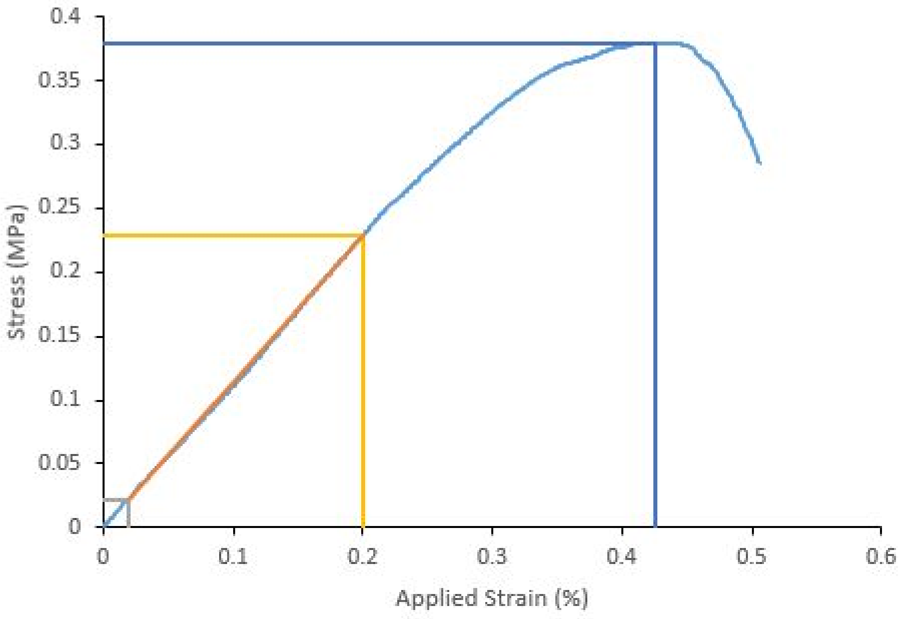

Figure S26. Representative stress strain curve for polymer 5.

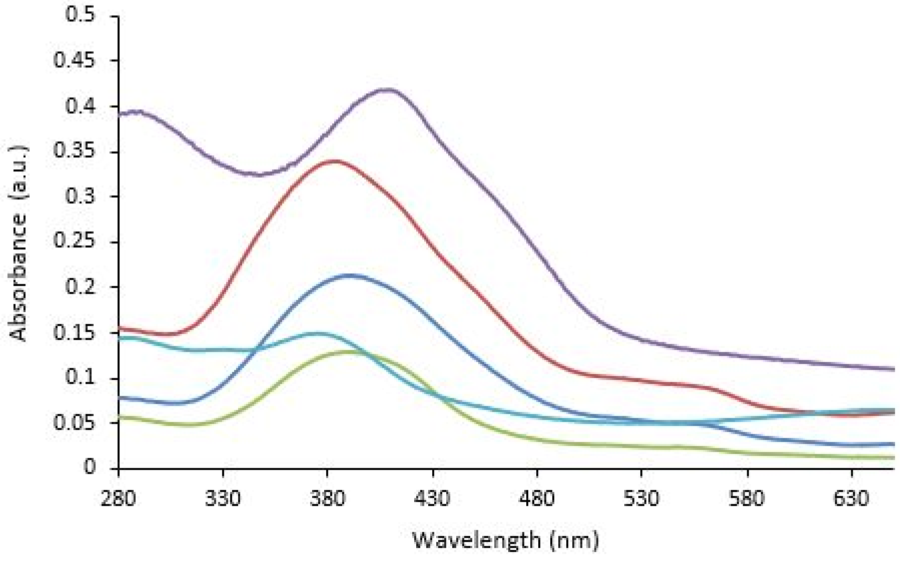

Figure S27. UV-Vis of polymers films cast on quartz slides. Polymer 1 - Blue; Polymer 2 - Red; Polymer 3 - Light blue; Polymer 4 - Purple; Polymer 5 - Green.

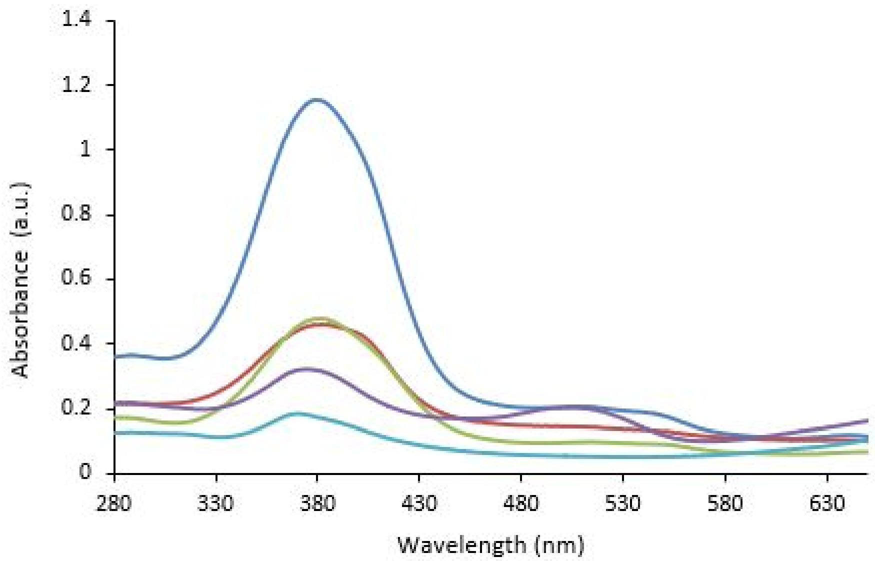

Figure S28. UV-Vis of polymers films and doped with CSA (10% by mass) cast on quartz slides. Polymer 1 - Blue; Polymer 2 - Red; Polymer 3 - Light blue; Polymer 4 - Purple; Polymer 5 - Green.

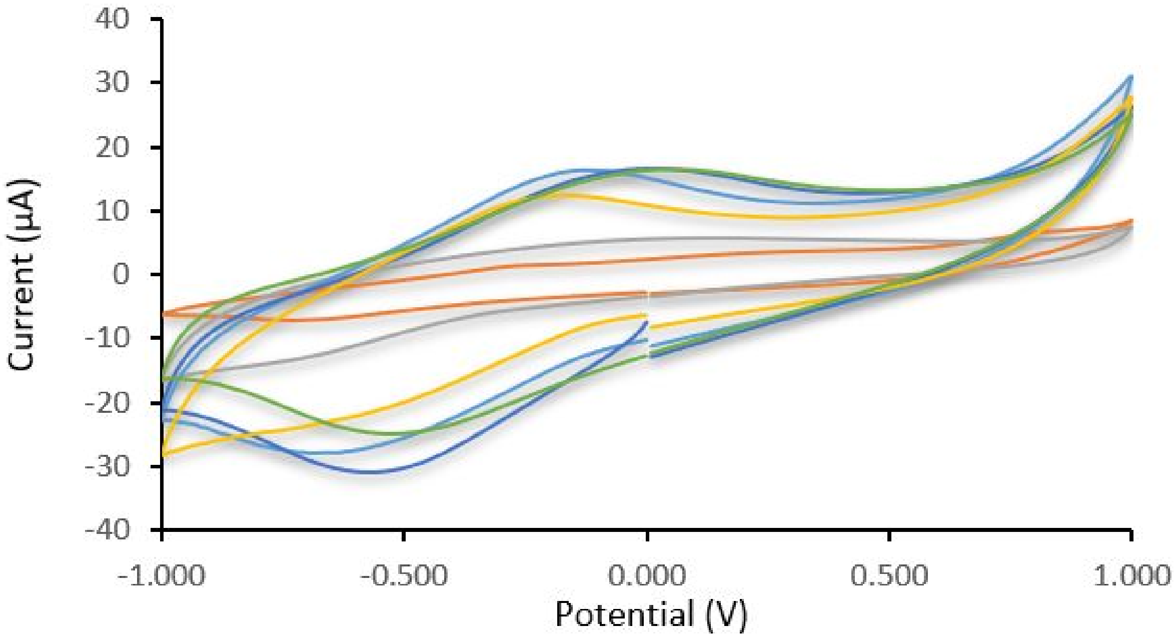

Figure S29. Voltammograms of polymer films on glassy carbon electrodes in PBS. Polymer 1 (dark blue), polymer 2 (yellow), polymer 3 (green), polymer 4 (grey) and polymer 5 (light blue) versus the no-polymer blank control (orange). Scan rate = 50 mV s^{-1}.

Table S2. In silico toxicity prediction data for polymers 1-5.

Compound	Endpoint	Reasoning level	Alert name	References	SMILES
Polymer 1	Standard	Plausible	Hepatotoxicity - Conjugated alkene	1 - 17	CC1=C(C=C)C2=[N]3/C1=C\C4=C(C=C)C(C)=C(/C=C5[N]6=C(C=C(C(CCC(OCCCCCC(OCCOCCOC(CCCCCO[H])=O)=O)=O)=C7C)N([Fe]36)C7=C2)C(CCC(OC)=O)=C\5C)N4C
Polymer 2	Standard	Plausible	Hepatotoxicity - Conjugated alkene	1 - 17	CC1=C(C=C)C2=[N]3C1=CC4=C(C=C)C(C)=C5N4[Fe]3(N6C(C(C)=C(CCC(OCCCCCC(OCCOCCOC(CCCCCO[H])=O)=O)=O)C6=C7)=C2)(Cl)[N]8=C7C(CCC(OCCCCCC(OCCOCCOC(CCCCCO[H])=O)=O)=O)=C(C)C8=C5
Polymer 3	Standard	Plausible	Hepatotoxicity - Conjugated alkene	1 - 17	CC1=C(C=C)C2=[N]3/C1=C\C4=C(C=C)C(C)=C(/C=C5[N]6=C(C=C(C(CCC(OCCCCC(OCC(O[R])(O[R])CC)=O)=O)=C7C)N([Fe]36)C7=C2)C(CCC(OC)=O)=C\5C)N4C
	Standard	Equivocal	Nephrotoxicity - Dihydroxypropane or derivative.	18	
Polymer 4	Standard	Plausible	Hepatotoxicity - Conjugated alkene	1 - 17	CC(C(N/1)=O)=C(C=C)C1=C\C2=C(C(CCC(OCCCCCC(OCCOCCOC(CCCCCOC)=O)=O)=O)=C(CC3=C(C(C)=C(/C=C4NC(C(C=C)=C\4C)=O)N3)CCC(OC)=O)N2)C

Polymer 5	Standard	Plausible	Hepatotoxicity - Conjugated alkene	1 - 17	CC(C(N/1)=O)=C(C=C)C1=C\C2=N/C(C(CCC(OCCCCCC(OCCOCCOC(CCCCCOC)=O)=O)=O)=C2C)=C\C3=C(C(C)=C(/C=C4NC(C(C=C)=C\4C)=O)N3)CCC(OC)=O
Hemin	Standard	Plausible	Hepatotoxicity - Conjugated alkene	4 - 17	CC1=C(C=C)C2=N/C1=C\C3=C(C=C)C(C)=C(/C=C4N=C5C(CCC(O)=O)=C\4C)N3[Fe](Cl)N6/C(C(C)=C(CCC(O)=O)/C6=C/5)=C\2
		Equivocal	Mitochondrial dysfunction	18 - 23	
Biliverdin	Standard	Plausible	Hepatotoxicity - Conjugated alkene	4 - 17	CC(C(N/1)=O)=C(C=C)C1=C\C2=C(C)C(CCC(O)=O)=C(/C=C3N=C(C(C)=C\3CCC(O)=O)/C=C(N4)/C(C)=C(C=C)C4=O)N2
		Equivocal	Mitochondrial dysfunction	18 - 23	
Bilirubin	Standard	Plausible	Hepatotoxicity - Conjugated alkene	4 - 17	CC(C(N/1)=O)=C(C=C)C1=C\C2=C(C)C(CCC(O)=O)=C(CC3=C(CCC(O)=O)C(C)=C(/C=C(N4)/C(C)=C(C=C)C4=O)N3)N2
		Equivocal	Mitochondrial dysfunction	18 - 23	
PCL Diol	Standard	N/A	N/A	N/A	[H]OCCCCCC(OCCOCCOC(CCCCCO[H])=O)=O
PCL Triol	Standard	Equivocal	Nephrotoxicity - Dihydroxypropane or derivative.	18	[*]C(CCCCCO[H])=O.CCC(CO[R])(CO[R])CO[R]

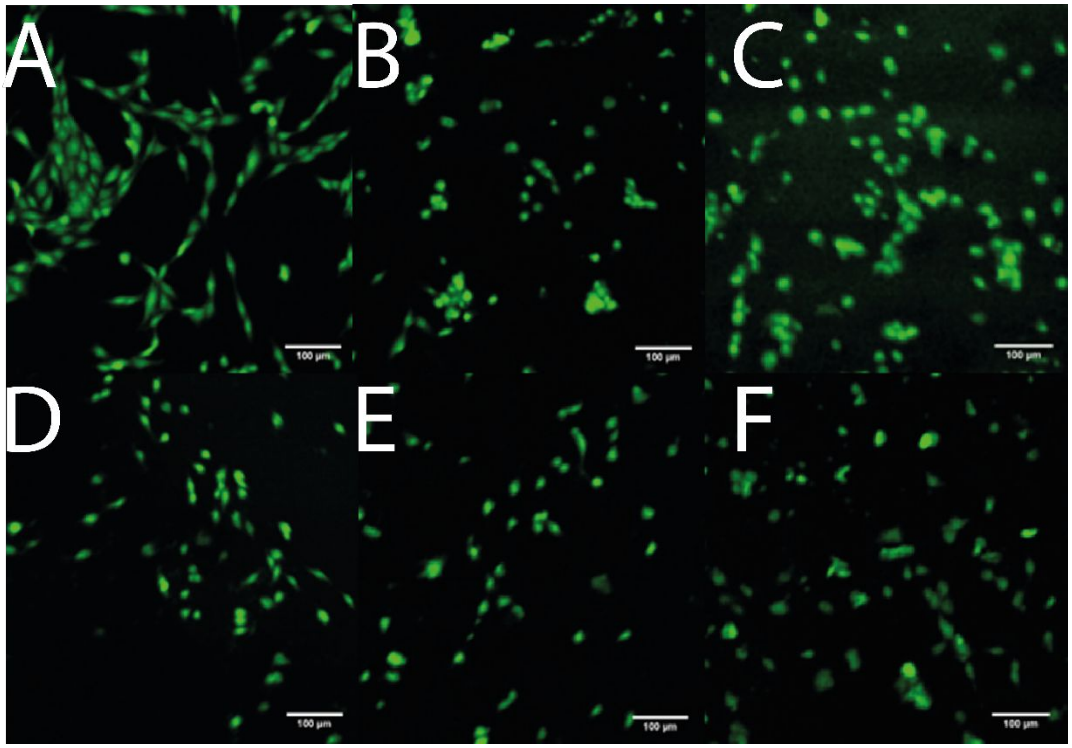

Figure S30. Images of cell adhesion on substrates (cells stained with Alexa Fluor™ 488 Phalloidin) after 24 hours at 20x magnification (scale bars represent 100 μm). **A)** control (tissue culture plastic). **B)** Polymer 1. **C)** Polymer 2. **D)** Polymer 3. **E)** Polymer 4. **F)** Polymer 5.

Table S3. In vitro cell viability of fibroblasts on polymers **1-5** at 1, 2, 3, 4 and 7 days relative to control (tissue culture plastic, 100%).

**Time (days)**	**Polymer 1**	**Polymer 2**	**Polymer 3**	**Polymer 4**	**Polymer 5**
1	51.0 ± 16.0 %	49.8 ± 12.0 %	46.7 ± 3.8 %	63.2 ± 16.0 %	37.1 ± 8.5 %
2	69.3 ± 10.9 %	61.8 ± 9.5 %	79.3 ± 14.0 %	70.3 ± 5.9 %	84.1 ± 7.9 %
3	62.5 ± 7.0 %	68.2 ± 8.4 %	77.3 ± 13.5 %	57.7 ± 5.3 %	78.5 ± 12.5 %
4	65.2 ± 8.8 %	51.3 ± 3.2 %	75.8 ± 4.1 %	55.2 ± 5.4 %	34.5 ± 17.5 %

7	48.2 ± 17.5 %	55.1 ± 2.9 %	69.5 ± 9.5 %	52.8 ± 7.5 %	45.5 ± 8.2 %

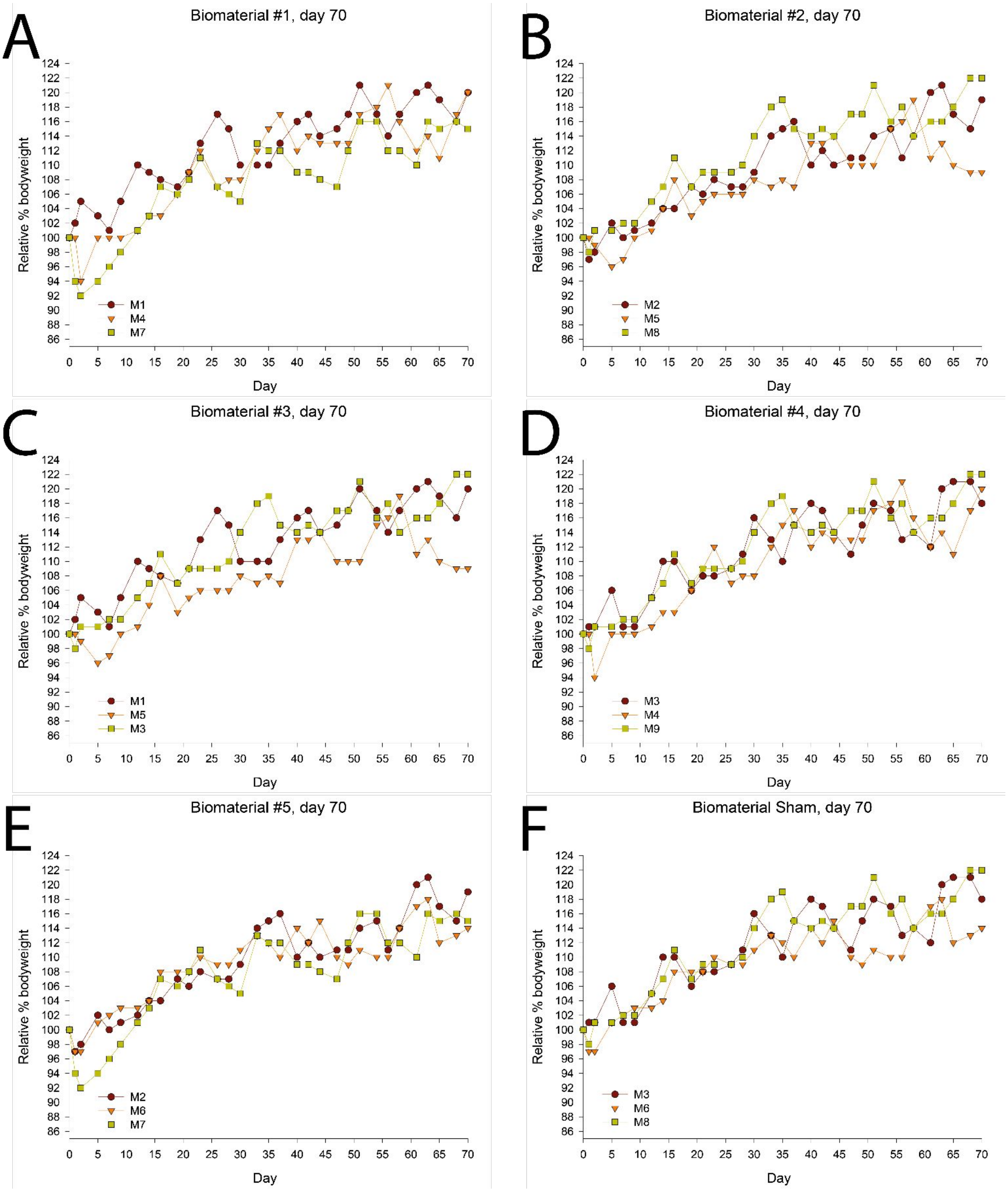

Figure S31. Analysis of body weight for mice over 70 days (no deleterious effects or unexpected fluctuations in body weight were observed during the study). **A)** Mice with films of polymer 1 implanted. **B)** Mice with films of polymer 2 implanted. **C)** Mice with films of polymer 3 implanted. **D)** Mice with

films of polymer 4 implanted. **E)** Mice with films of polymer 5 implanted. **F)** Mice with no films implanted (i.e. sham surgery).

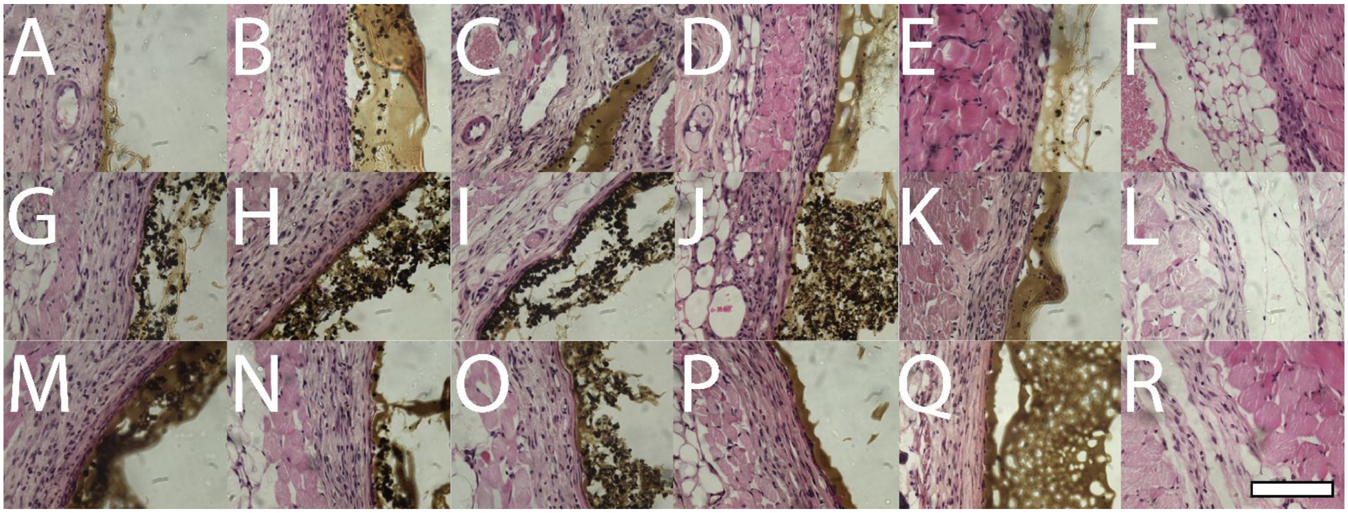

Figure S32. Images of the implant *in situ* in Balb/C mice culled at day 7 **(Images A-F)**, day 28 **(Images G-L)** or day 70 **(Images M-R)**. **A), G) and M)** Polymer 1. **B), H) and N)** Polymer 2. **C), I) and O)** Polymer 3. **D), J) and P)** Polymer 4. **E), K) and Q)** Polymer 5. **F), L) and R)** Sham surgery control – no film implanted. There was no evidence of inflammation surrounding the implants was seen upon gross examination post-mortem. The 'flaking' surrounding the incision on day 7 is not due to the presence of the material, but is instead due to the tissue glue used to bond the incision, which healed within 2 weeks and was no longer evident at later time points. Scale bar represents 10 mm.

Table S4. Blood count data after implantation of films of polymers **1-5**.

	Lymphocytes			Neutrophils			Other (Monocytes plus Eosino-/Baso-phils)		
	La	Lb	Lc	Na	Nb	Nc	Ma	Mb	Mc
1 day 7	73	69	73	25	26	22	2	5	5
1 day 28	65	72	66	31	26	30	4	2	4
1 day 70	68	73	55	27	23	42	5	4	3
2 day 7	73	59	70	25	34	27	2	7	3
2 day 28	76	70	66	19	26	30	5	4	4
2 day 70	72	69	74	25	28	24	3	3	2
3 day 7	74	69	70	24	26	27	2	5	3
3 day 28	70	74	73	27	23	24	3	3	3
3 day 70	68	69	62	27	28	35	5	3	3
4 day 7	54	64	73	41	31	22	5	5	5
4 day 28	70	70	77	27	26	19	3	4	4
4 day 70	75	73	62	22	23	35	3	4	3
5 day 7	54	59	63	41	34	34	5	7	3
5 day 28	65	74	77	31	23	19	4	3	4
5 day 70	72	56	55	25	41	42	3	3	3
sh day 7	74	64	63	24	31	34	2	5	3
sh day 28	76	72	73	19	26	24	5	2	3
sh day 70	75	56	74	22	41	24	3	3	2
(red)	Lymphocytes <65%								
(green)	Neutrophils >30%								
(yellow)	Others >5%								

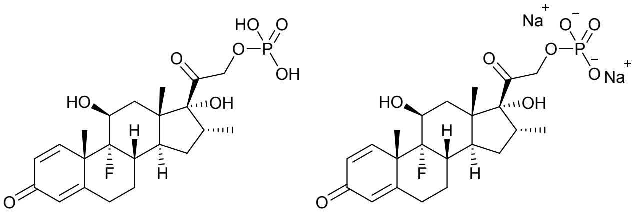

Figure S33. Chemical structure of DMP (left) and the corresponding disodium salt derivative (right).

References

(1) Lorimer, W. V.; Lilis, R.; Fischbein, A.; Daum, S.; Anderson, H.; Wolff, M. S.; Selikoff, I. J. Health Status of Styrene-Polystyrene Polymerization Workers. *Scand. J. Work. Environ. Health* **1978**, *4* (2), 220–226. https://doi.org/10.5271/sjweh.2743.

(2) Program, N. T. NTP Toxicology and Carcinogenesis Studies of 1,3-Butadiene (CAS No. 106-99-0) in B6C3F1 Mice (Inhalation Studies). *Natl. Toxicol. Program Tech. Rep. Ser.* **1993**, *467* (1), 1–379.

(3) Sinha, A. K.; Joshi, B. P.; Dogra, R. One Step Conversion of Toxic β-Asarone from Acorus

Calamus into 1-(2,4,5-Trimethoxyphenyl)-1,2-Dihydroxypropane and Asaronaldehyde Occurring in Piper Clusii. *Nat. Prod. Lett.* **2001**, *15* (6), 439–444. https://doi.org/10.1080/10575630108041315.

(4) MORGAN, D. L.; MAHLER, J. F.; O'CONNOR, R. W.; PRICE, H. C.; ADKINS, B. Styrene Inhalation Toxicity Studies in Mice. *Toxicol. Sci.* **1993**, *20* (3), 325–335. https://doi.org/10.1093/toxsci/20.3.325.

(5) Dahl, S. L.; Ward, J. R. Pharmacology, Clinical Efficacy, and Adverse Effects of the Nonsteroidal Anti-Inflammatory Agent Benoxaprofen. *Pharmacother. J. Hum. Pharmacol. Drug Ther.* **1982**, *2* (6), 354–365. https://doi.org/10.1002/j.1875-9114.1982.tb03212.x.

(6) Chatila, R. Hepatotoxicity: The Adverse Effects of Drugs and Other Chemicals on the Liver,. *J. Clin. Gastroenterol.* **2000**, *31* (2), 185. https://doi.org/10.1097/00004836-200009000-00024.

(7) Dong, J. Q.; Liu, J.; Smith, P. C. Role of Benoxaprofen and Flunoxaprofen Acyl Glucuronides in Covalent Binding to Rat Plasma and Liver Proteins in Vivo. *Biochem. Pharmacol.* **2005**, *70* (6), 937–948. https://doi.org/10.1016/j.bcp.2005.05.026.

(8) Peters, T. S. Do Preclinical Testing Strategies Help Predict Human Hepatotoxic Potentials? *Toxicol. Pathol.* **2005**, *33* (1), 146–154. https://doi.org/10.1080/01926230590522121.

(9) El Sawy, N. A.; El-Zwahry, A. M.; Attia, L. A. Influence of Two Non-Steroidal Anti-Inflammatory Drugs on Some Biochemical Parameters in Arthritic Male Albino Rats. *Egypt. J. Biomed. Sci.* **2001**, *34* (6), 219–235.

(10) Wade, L. T.; Kenna, J. G.; Caldwell, J. Immunochemical Identification of Mouse Hepatic Protein Adducts Derived from the Nonsteroidal Anti-Inflammatory Drugs Diclofenac, Sulindac, and Ibuprofen. *Chem. Res. Toxicol.* **1997**, *10* (5), 546–555. https://doi.org/10.1021/tx960153t.

(11) Falzon, M.; Whiting, P. H.; Ewen, S. W. B.; Milton, A. S.; Burke, M. D. Comparative Effects of Indomethacin on Hepatic Enzymes and Histology and on Serum Indices of Liver and Kidney Function in the Rat. *Br. J. Exp. Pathol.* **1985**, *66* (5), 527–534.

(12) Wang, M.; Dickinson, R. G. Bile Duct Ligation Promotes Covalent Drug-Protein Adduct Formation in Plasma but Not in Liver of Rats given Zomepirac. *Life Sci.* **2000**, *68* (5), 525–537. https://doi.org/10.1016/S0024-3205(00)00958-9.

(13) Kethu, S. R.; Rukkannagari, S.; Lansford, C. L. Oxaprozin-Induced Symptomatic Hepatotoxicity. *Ann. Pharmacother.* **1999**, *33* (9), 942–944. https://doi.org/10.1345/aph.18408.

(14) Bailey, M. J.; Dickinson, R. G. Acyl Glucuronide Reactivity in Perspective: Biological Consequences. *Chem. Biol. Interact.* **2003**, *145* (2), 117–137. https://doi.org/10.1016/S0009-2797(03)00020-6.

(15) MacPhail, C. M.; Lappin, M. R.; Meyer, D. J.; Smith, S. G.; Webster, C. R. L.; Armstrong, P. J. Hepatocellular Toxicosis Associated with Administration of Carprofen in 21 Dogs. *J. Am. Vet. Med. Assoc.* **1998**, *12* (1), 885–901.

(16) Boelsterli, U. Diclofenac-Induced Liver Injury: A Paradigm of Idiosyncratic Drug Toxicity. *Toxicol. Appl. Pharmacol.* **2003**, *192* (3), 307–322. https://doi.org/10.1016/S0041-008X(03)00368-5.

(17) Mamou, P.; Levy, V. G. [Liver Damage Following Clomethacin Treatment. 6 Cases, Including 2

Deaths (Author's Transl)]. *Nouv. Presse Med.* **1981**, *33* (10), 2719–2722.

(18) Zhang, H.; Chen, Q.-Y.; Xiang, M.-L.; Ma, C.-Y.; Huang, Q.; Yang, S.-Y. In Silico Prediction of Mitochondrial Toxicity by Using GA-CG-SVM Approach. *Toxicol. Vitr.* **2009**, *23* (1), 134–140. https://doi.org/10.1016/j.tiv.2008.09.017.

(19) Geneve, J.; Hayat-Bonan, B.; Labbe, G.; Degott, C.; Letteron, P.; Freneaux, E.; Dinh, T. L.; Larrey, D.; Pessayre, D. Inhibition of Mitochondrial Beta-Oxidation of Fatty Acids by Pirprofen. Role in Microvesicular Steatosis Due to This Nonsteroidal Anti-Inflammatory Drug. *J. Pharmacol. Exp. Ther.* **1987**, *242* (3), 1133–1137.

(20) Varga, Z. V.; Ferdinandy, P.; Liaudet, L.; Pacher, P. Drug-Induced Mitochondrial Dysfunction and Cardiotoxicity. *Am. J. Physiol. Circ. Physiol.* **2015**, *309* (9), 1453–1467. https://doi.org/10.1152/ajpheart.00554.2015.

(21) Kirkman, S. K.; Zhang, M. Y.; Horwatt, P. M.; Scatina, J. Isolation and Identification of Bromfenac Glucoside from Rat Bile. *Drug Metab. Dispos.* **1998**, *26* (7), 720–723.

(22) Freneaux, E.; Fromently, B.; Berson, A.; Labbe, G.; Degott, C.; Letteron, P.; Larrey, D.; Pessayre, D. Stereoselective and Nonstereoselective Effects of Ibuprofen Enantiomers on Mitochondrial β-Oxidation of Fatty Acids. *J. Pharmacol. Exp. Ther.* **1990**, *255* (2), 529–535.

(23) Li, C.; Grillo, M. P.; Benet, L. Z. In Vivo Mechanistic Studies on the Metabolic Activation of 2-Phenylpropionic Acid in Rat. *J. Pharmacol. Exp. Ther.* **2003**, *305* (1), 250–256. https://doi.org/10.1124/jpet.102.043174.
