# Supplementary material for: Correction to “Controlled Bioactive Delivery Using Degradable Electroactive Polymers”
Source: Biomacromolecules. 2025 Sep 15;26(10):7221. doi: 10.1021/acs.biomac.5c00295 (PMC12522129; doi:10.1021/acs.biomac.5c00295)
Supplement: Supplementary file 1 [file bm5c00295_si_001.pdf]

Correction to “Controlled bioactive delivery using degradable electroactive polymers”

Mark D. Ashton, Patricia A. Cooper, Sofia Municoy, Martin F. Desimone, David Cheneler, Steven D. Shnyder and John G. Hardy*

Biomacromolecules 2022, 23, 7, 3031–3040.
https://doi.org/10.1021/acs.biomac.2c00516

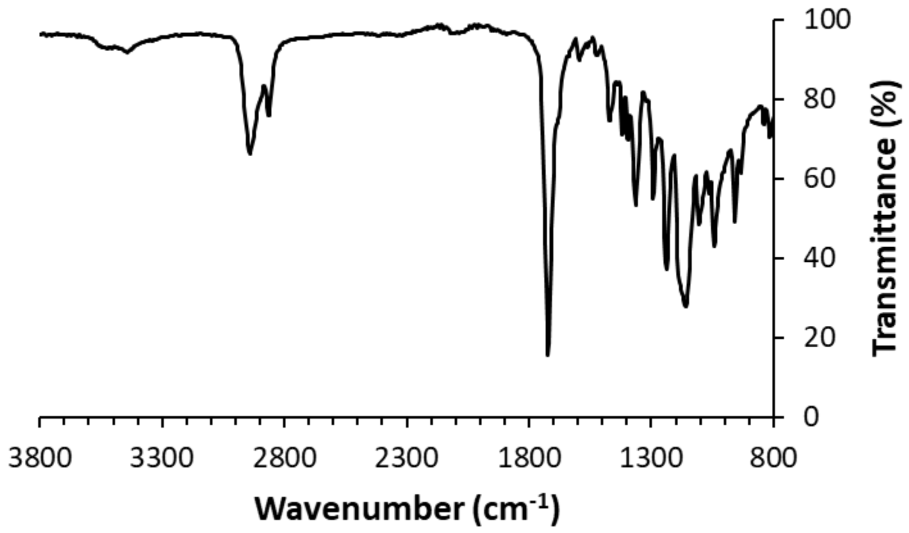

Figure S1. IR spectrum of alcohol-terminated PCL Diol 2000 g.mol^{-1}.

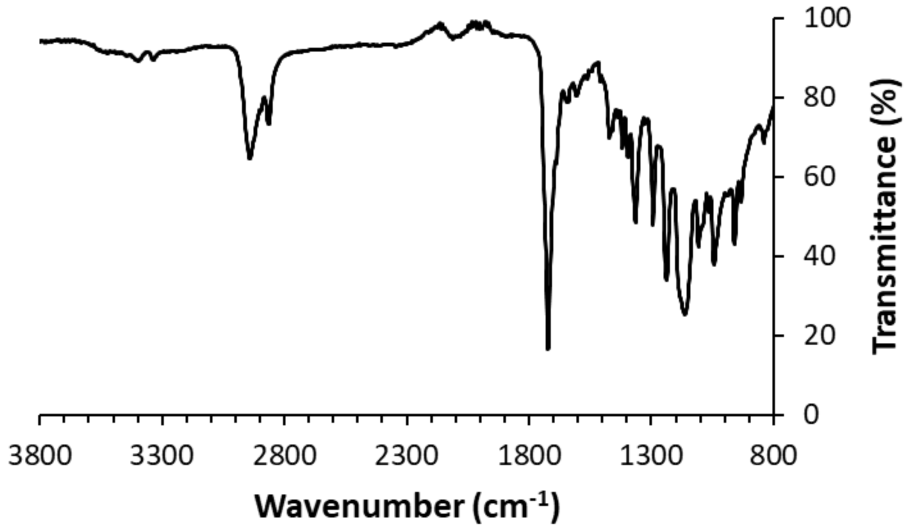

Figure S2. IR spectrum of PCL Diol 530 g.mol^{-1}.

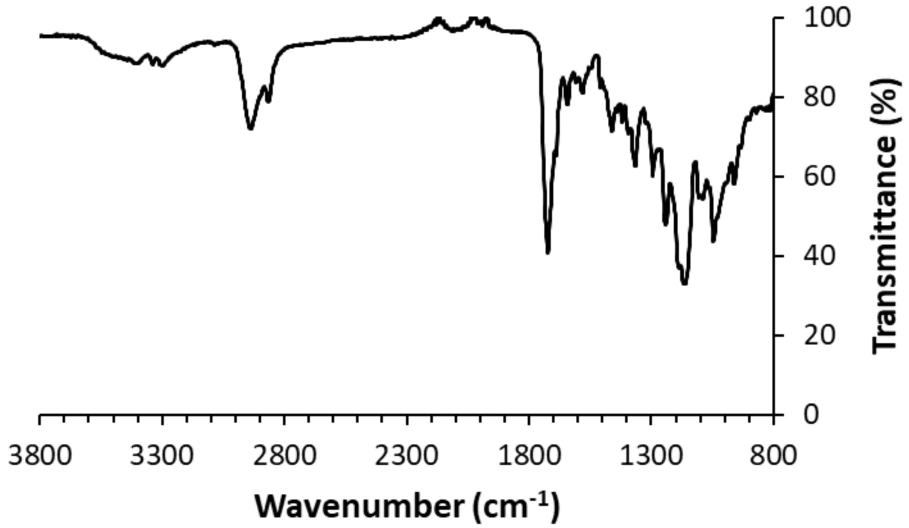

Figure S3. IR spectrum of PCL Triol 900 g.mol^{-1}.

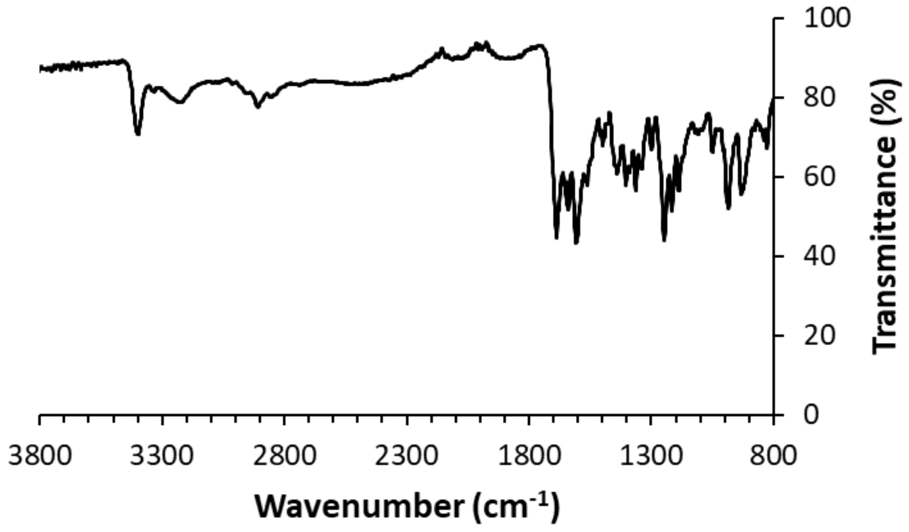

Figure S4. IR spectrum of Bilirubin.

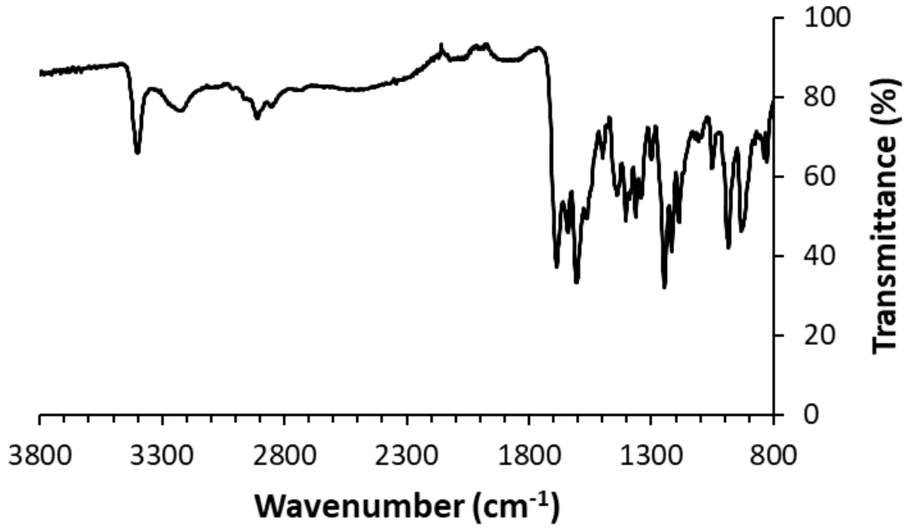

Figure S5. IR spectrum of Biliverdin.

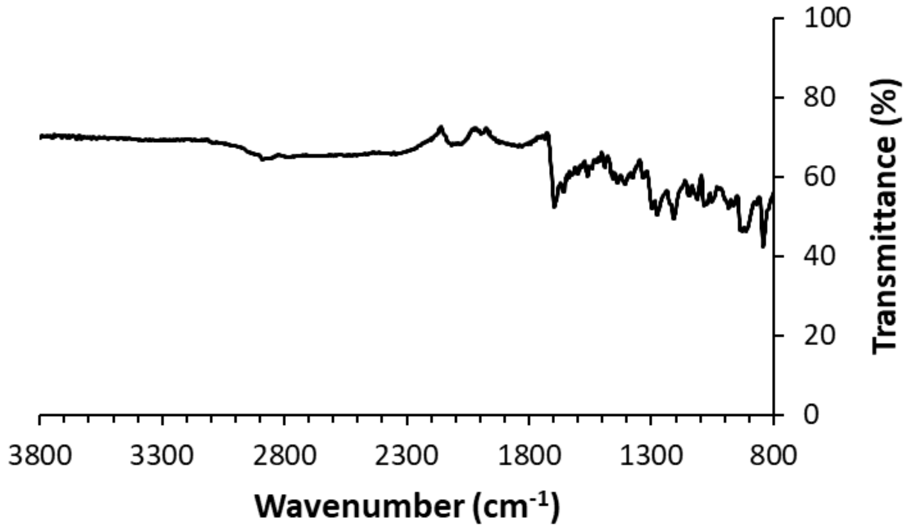

Figure S6. IR spectrum of hemin.

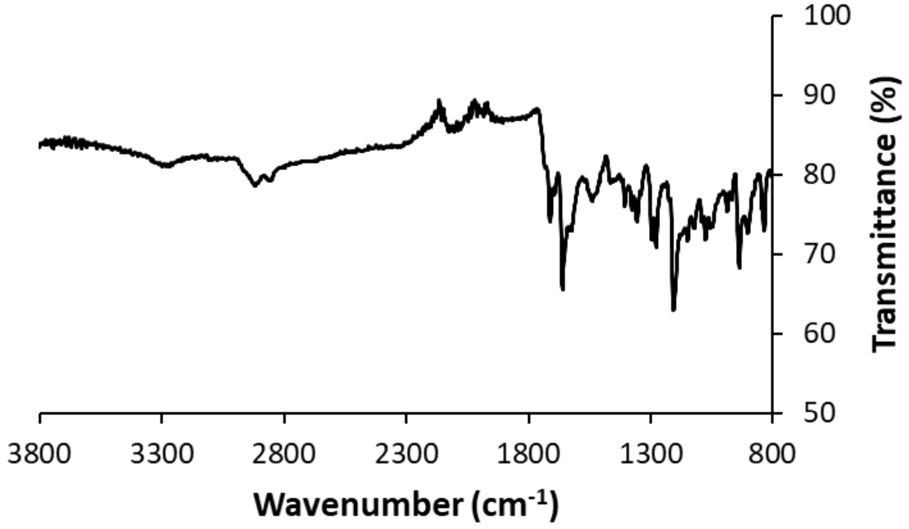

Figure S7. IR spectrum of polymer 1.

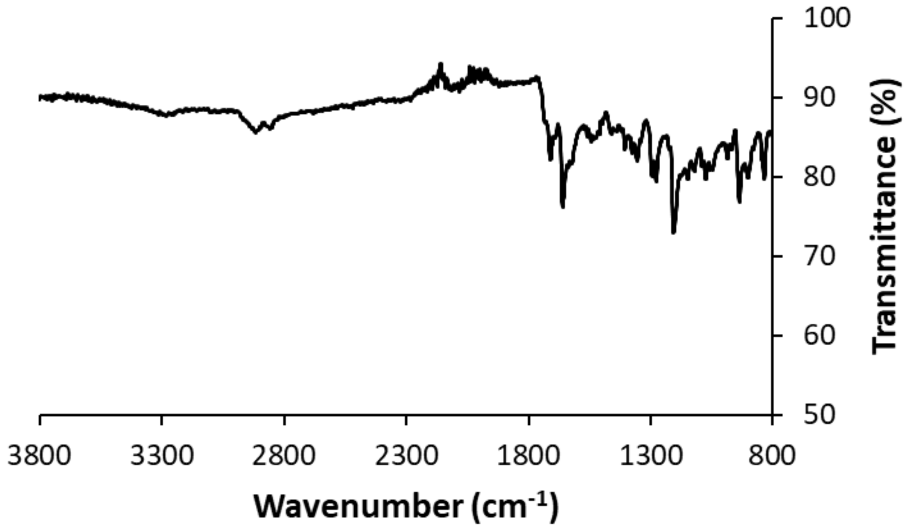

Figure S8. IR spectrum of polymer 2.

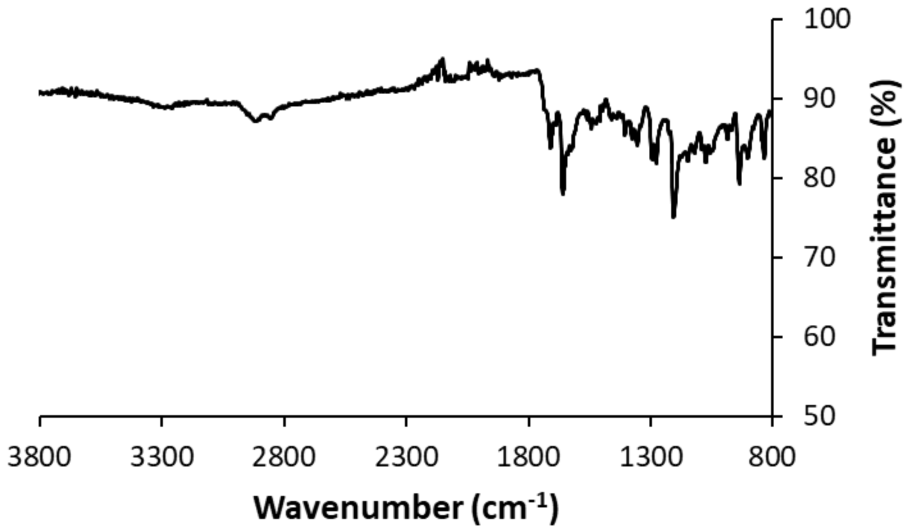

Figure S9. IR spectrum of polymer 3.

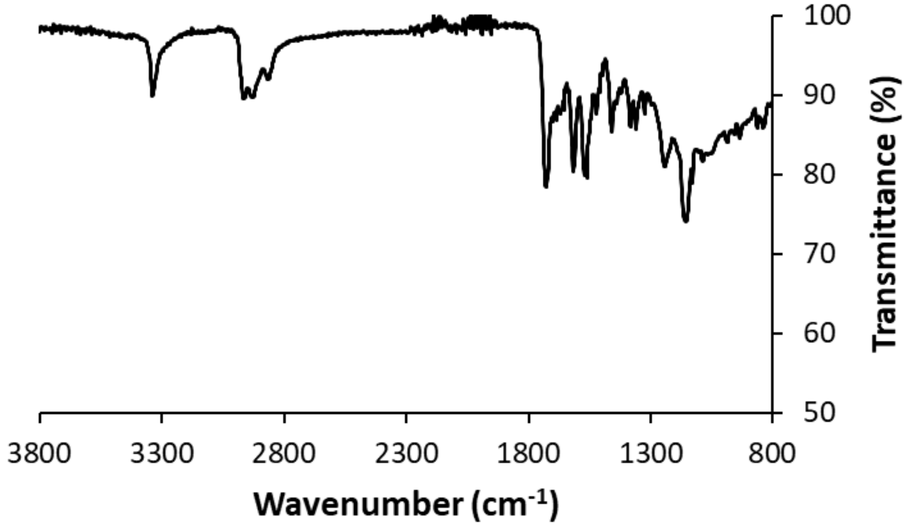

Figure S10. IR spectrum of polymer 4.

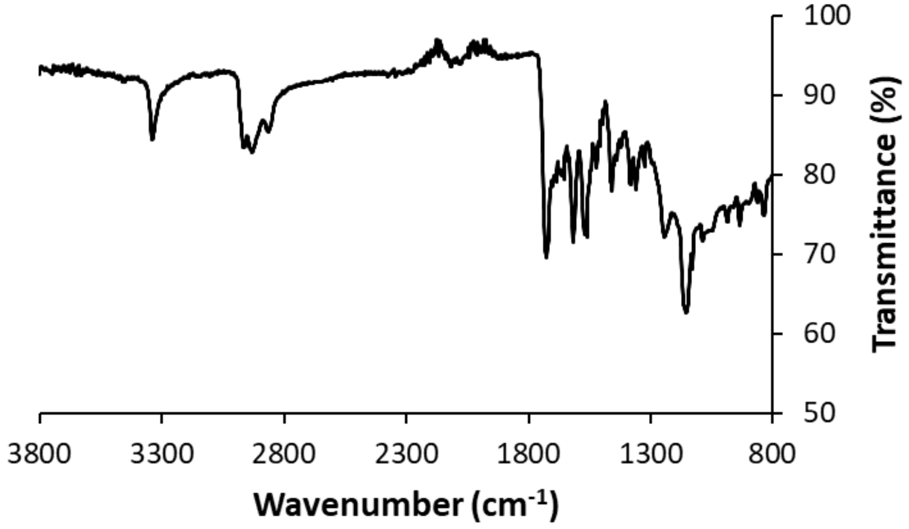

Figure S11. IR spectrum of polymer 5.
